# Diagnostic Twins: Exploring the Radiohybrid Concept with Iodine-123 and Lanthanum-133 for PSMA-Targeted SPECT and PET Imaging

**DOI:** 10.1021/acs.jmedchem.6c00161

**Published:** 2026-04-16

**Authors:** Tobias Krönke, Martin Ullrich, Magdalena K. Blei, Kristof Zarschler, Jonas Schädlich, Santiago Andrés Brühlmann, Klaus Kopka, Jens Pietzsch, Sven Stadlbauer, Constantin Mamat

**Affiliations:** † 9151 Helmholtz-Zentrum Dresden-Rossendorf, Institute of Radiopharmaceutical Cancer Research, Bautzner Landstraße 400, D-01328 Dresden, Germany; ‡ TU Dresden, School of Science, Faculty of Chemistry and Food Chemistry, D-01062 Dresden, Germany; § National Center for Tumor Diseases (NCT), NCT/UCC Dresden, a Partnership Between DKFZ, Faculty of Medicine and University Hospital Carl Gustav Carus, TU Dresden & Helmholtz-Zentrum Dresden-Rossendorf (HZDR), D-01307 Dresden, Germany; ∥ German Cancer Consortium (DKTK), Partner Site Dresden, and German Cancer Research Center (DKFZ), D-69120 Heidelberg, Germany

## Abstract

PSMA-targeted ligands containing macropa as highly effective chelators for ^225^Ac were developed for endoradiotherapy, alongside complementary diagnostic approaches using ^133^La for PET and ^123^I for SPECT within the radiohybrid concept. These ligands include mono- and bivalent PSMA-targeting structures with optional albumin-binding moieties, enabling both early- and late-stage imaging while maintaining identical pharmacological behavior. Radiolabeling was performed at the macropa side with ^133^La and at the albumin-binding side with ^123^I. All ligands showed high PSMA affinity (*K*_i_ = 2.3–9.4 nM) and remarkable internalization rates up to 97% in vitro. In vivo studies using LNCaP tumor-bearing mice demonstrated comparable tumor uptake across all conjugates, regardless of the radionuclide. Advantageously, quantitative PET and SPECT blood measurements correlated closely with ex vivo data and metabolite analysis, additionally confirming the high in vivo stability with minimal deiodination during renal excretion and highlighting the suitability for dosimetric applications.

## Graphical abstract

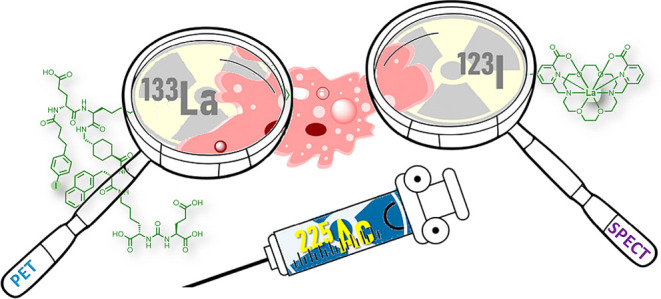

## Introduction

The management of advanced prostate cancer remains a significant challenge due to limited treatment options and poor prognosis associated with castration-resistant prostate cancer (CRPC), despite ongoing therapeutic advancements.^1^ The overexpression of prostate-specific membrane antigen (PSMA) on tumor cells has stimulated the development of radiopharmaceuticals in the treatment of mCRPC, thereby facilitating selective and specific targeting and localized systemic internal radiation delivery, also called molecular radiotherapy or targeted endoradiotherapy.^2^ Therapeutic radiopharmaceuticals, particularly based on alpha emitters, have emerged as beneficial in this context. Clinical studies have demonstrated that PSMA-targeted alpha therapy utilizing ^225^Ac can confer significant therapeutic benefits, including tumor shrinkage, pain relief, and enhanced survival outcomes in mCRPC patients, particularly those which no longer respond to or have experienced treatment failure with conventional modalities such as chemotherapy, immunotherapy, and second-line hormonal treatments.^3–5^ Notwithstanding the encouraging outcomes observed thus far, the clinical utility of ^225^Ac-based therapies remains contingent upon the availability and combination of sensitive and reliable diagnostic tools.

The primary diagnostic modality is positron emission tomography (PET) using PSMA-targeting tracers containing ^68^Ga or ^18^F, which have demonstrated high sensitivity in detecting both primary and metastatic lesions in mCRPC.^6^ The aforementioned imaging agents facilitate not only the selection of patients for treatment but also the monitoring of the therapy effects such as early detection of disease progression. However, they differ in their pharmacokinetic behavior compared to the appropriate therapeutic counterpart (e.g., [^177^Lu]­Lu-PSMA-617). On that account, there is a continued requirement for the development of radiotracers with improved pharmacokinetics that offer enhanced resolution, sensitivity, and broader clinical applicability.^7^

A significant drawback of the current radiopharmaceutical approach is the difficulty in accurately categorizing patients and the intricacy of monitoring treatment outcomes.^8^ The development of next-generation diagnostic radiotracers, which may provide superior tumor-to-background ratios, could enhance the efficacy of alpha therapy and expand its clinical utility. While the combination of PSMA-targeted alpha therapy with ^225^Ac offers a promising approach to treating mCRPC, its full clinical potential can only be realized through concurrent advances in diagnostic imaging.^9,10^ The majority of ^225^Ac-based radioconjugates is based on 1,4,7,10-tetraazacyclododecane-1,4,7,10-tetraacetic acid (DOTA) as a chelator.^3^ However, there are several reports, demonstrating that DOTA seems to be not ideal in the case of radiolabeling and stability.^11^ These obstacles can be improved by the use of macropa (mcp).^12^

The radiohybrid approach allows the use of radionuclides that have no diagnostic or therapeutic counterpart of the same element to be combined into a true matched pair.^13^ In this way, nuclides and radionuclides of different elements can be paired without altering the chemical structure of the tracer molecule and thus the pharmacokinetic properties. Most known radiohybrid ligands are based on the use of ^18^F together with ^177^Lu to obtain a radiohalogen-based tracer for noninvasive molecular imaging and a radiometal-based therapeutic radioligand that do not differ in their biodistribution.^14–18^ By using iodine-123 for molecular imaging and actinium-225 for targeted alpha therapy (TAT), we suggest here a new radiohybrid pair.

Iodine-123 is an excellent diagnostic radionuclide widely used in nuclear medicine for single-photon emission computed tomography (SPECT).^19^ With a half-life of 13.2 h, it provides an ideal balance between imaging flexibility and patient safety.^20^ ^123^I emits photons with an energy of 159 keV, which is well suited for high-resolution SPECT imaging systems, providing superior image quality while minimizing scattering effects.^21^ These properties make ^123^I highly effective for diagnostic purposes. Its versatility and favorable physical properties have made ^123^I a cornerstone of nuclear imaging.^22^

As the imaging capabilities of actinium radioisotopes are inadequate,^23^ lanthanum-133 is emerging as a promising diagnostic surrogate for the alpha-emitting therapeutic radionuclide actinium-225.^24,25^ Due to its chemical similarity to actinium (ion radius and charge), ^133^La provides a reliable prediction of the biodistribution and tumor targeting of ^225^Ac in both preclinical and clinical applications.^24–26^ Its half-life of 3.9 h is sufficient for effective PET imaging while ensuring low radiation exposure to patients.^27^ ^133^La is cyclotron-produced and emits low-energy positrons (*E*_β,mean_ = 463 keV), ensuring low radiation exposure to patients and allowing a precise tumor localization with exceptional spatial resolution.^27,28^

Building on macropa-based albumin-binding PSMA-targeting ligands that were previously developed for targeted alpha therapy with ^225^Ac, a need for corresponding diagnostic counterparts arose.^29,30^ The objective was to create diagnostic agents with identical pharmacological behavior that would enable noninvasive imaging techniques to be used, mirroring the biodistribution patterns observed for therapeutic applications. This would ensure a seamless transition between diagnosis and therapy. The present work introduces a theranostic approach including the development of SPECT and PET tracers, which are capable of being radiolabeled with cyclotron-produced radionuclides. To achieve this objective, a dual conception was employed, comprising the use of the β^+^-emitter lanthanum-133 to mimic the pharmacokinetics and act as a direct PET-diagnostic surrogate for actinium-225 and the use of the γ-emitter iodine-123 for SPECT imaging in a newly developed radiohybrid strategy,^13^ ideally using the albumin binder 4-(4-iodophenyl)­butyrate (abbreviated as **alb** and used in the compound name).^31^ While albumin binding may enhance tumor uptake through an extended blood residence, a prolonged circulation can also increase background activity and lead to higher accumulation in nontarget organs. This consideration is particularly relevant for alpha-emitting radionuclides due to their potential off-target toxicity.

So far, it is the first reported radiohybrid combination of ^123^I/^225^Ac within a theranostic concept. Advantageously, the combination of both diagnostic radionuclides allows a precise diagnosis at early imaging time points via PET in the case of ^133^La as well as long-term imaging possibilities in the case of ^123^I, which is important even to monitor the biodistribution behavior as a prerequisite for the ^225^Ac-therapy. Additionally, it should serve to improve dosimetry calculations for a target-oriented alpha therapy due to the approximately 3-fold longer half-life of ^123^I compared to ^133^La.

The influence of the metal load in the chelator of the radioconjugates on the binding affinity and biodistribution was explored. Detailed *in vivo* experiments using LNCaP tumor-bearing mice were accomplished including quantitative PET and SPECT imaging, blood kinetics, biodistribution, and metabolite analyses. For completion, blood activity concentrations were validated against *ex vivo* blood sampling.

## Results and Discussion

Due to the use of a (radio)­halogen in combination with a (radio)­metal, the synthesis effort for the radiohybrid concept is higher in general.^13^ This includes the synthesis of the macropa-based conjugate for radiometal labeling as well as a precursor conjugate with a leaving group to connect the radiohalogen. At this point, the systematic investigation of this concept includes the influence of the radiolabel position (radioiodine on the albumin binder vs. radiometal in the chelator), which could affect the biodistribution. To this end, variants of the same scaffold were synthesized with both (radio)­iodinated and (radio)metal labels. The PSMA-617-derived binding motif was synthesized as previously published^29,30^ to generate the monovalent, bispecific **mcp-M-alb-PSMA (3)** and the bivalent, bispecific **mcp-D-alb-PSMA (4)** using the macropa-based building blocks **mcp-M-click (1)** and **mcp-D-click (2)** (Figure 1).

**Figure 1:**
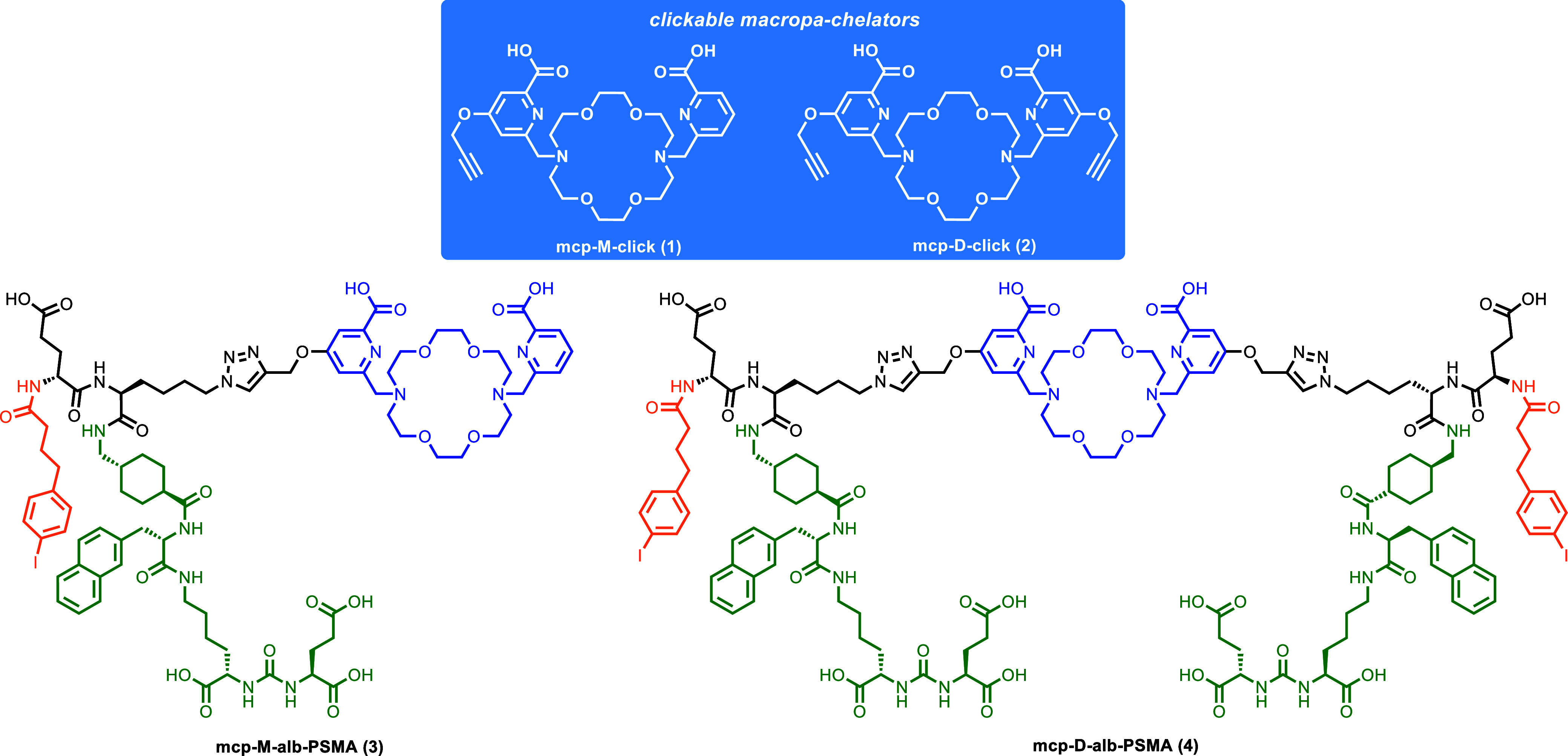
Structures of **mcp-M-alb-PSMA (3)** and **mcp-D-alb-PSMA (4)** with macropa (blue), albumin binder (orange) and PSMA-binding motif (green), and their respective macropa-building blocks **mcp-M-click (1)** and **mcp-D-click (2)** (blue box).^37,38^

To perform the radiolabeling with ^123^I, two new precursor compounds **mcp-M-Sn-alb-PSMA** and **mcp-D-Sn-alb-PSMA** with trimethylstannyl leaving groups were established, allowing the electrophilic introduction of radioiodine under mild conditions. The synthesis of the two precursors was designed to yield two new ^123^I-radiotracers that retain the albumin-binding properties through the 4-(*p*-iodophenyl) butyrate (**alb**) moiety while simultaneously enabling noninvasive molecular SPECT imaging. To achieve this, the activated ester 4-nitrophenyl 4-(4-(trimethylstannyl)­phenyl)­butanoate (**7**) was prepared from the carboxylic acid **6**,^32^ which was prepared via a palladium-catalyzed iodine-tin-exchange reaction from compound **5** (Scheme 1).

**Scheme 1:**
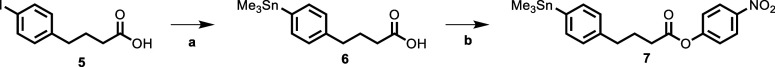
Synthesis of the Stannylated Precursor Building Block **7** ^a^

a Reaction conditions: (a) hexamethylditin, tetrakis­(triphenylphosphane)­palladium(0), toluene, 100 °C, 4 h; (b) *p*-nitrophenol, dicyclohexylcarbodiimide, dichloromethane, rt, overnight (see supporting information for synthesis details).

To prepare the PSMA-binding unit, the established synthesis pathway leading to compound **8** was adapted.^29^ In this procedure, PSMA-compound **8** was connected to the macropa chelator (either **mcp-M-click (1)** or **mcp-D-click (2)**) via copper-catalyzed azide–alkyne cycloaddition to obtain compounds **9** and **11**. The final compounds **10** and **12** ready for electrophilic radioiodination were obtained through the reaction of **9** and **11**, respectively, with *p*-nitrophenyl active ester **7**. The complete reaction is shown in Scheme 2. Due to the limited stability of the trimethylstannyl group in an acidic environment, no additional purification was done for **mcp-M-Sn-alb-PSMA** and **mcp-D-Sn-alb-PSMA**.

**Scheme 2:**
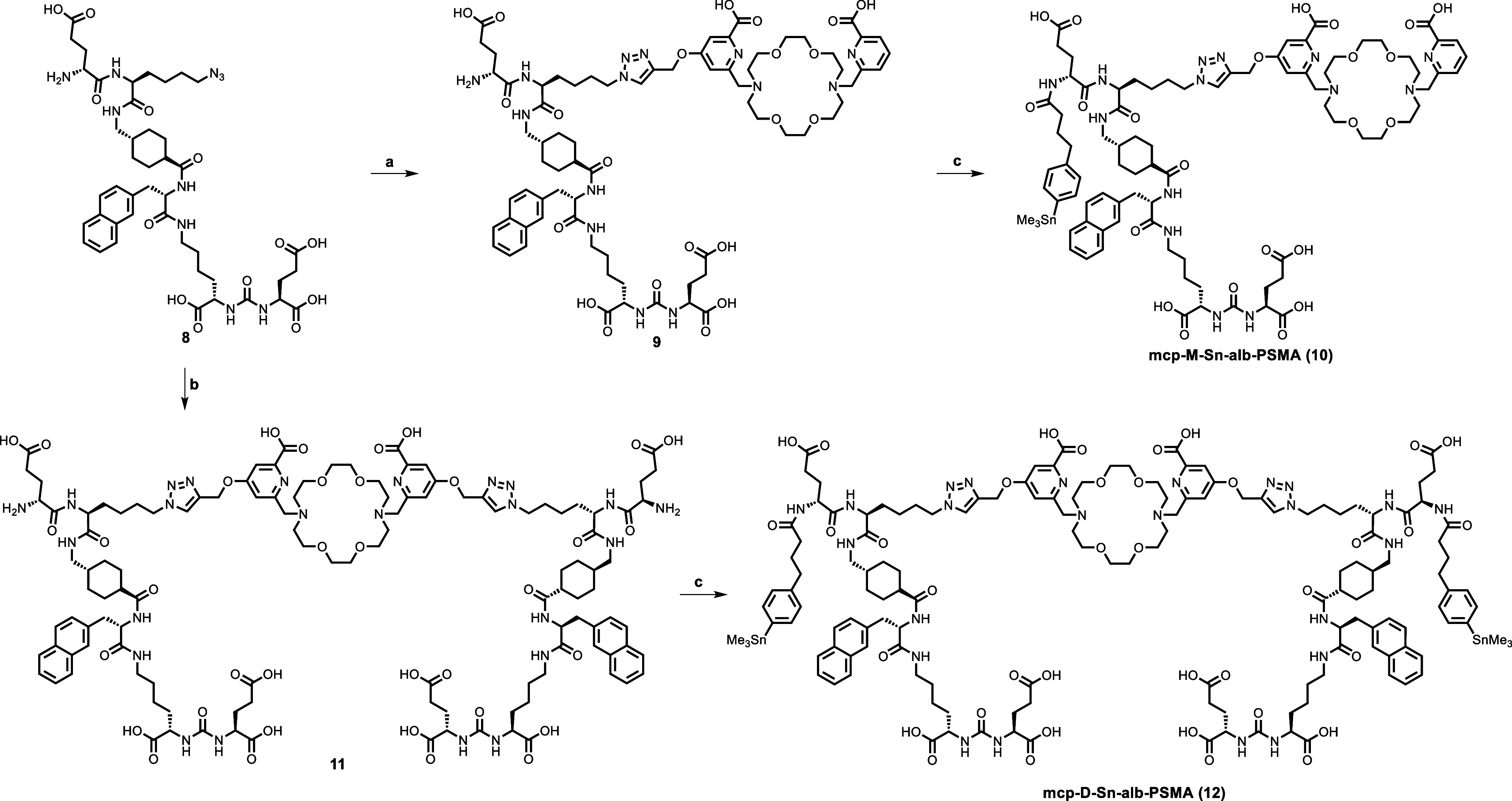
Synthesis of Two Precursors **mcp-M-Sn-alb-PSMA (10)** and **mcp-D-Sn-alb-PSMA (12)** for Radiolabeling With ^123^I^a^

a Reaction conditions: (a) **mcp-M-click (1)**, CuSO_4_·5 H_2_O, tris­(3-hydroxypropyltriazolylmethyl)­amine, sodium (l)-ascorbate, t-BuOH, H_2_O, rt, overnight; (b) **mcp-D-click (2)**, CuSO_4_·5 H_2_O, tris­(3-hydroxypropyltriazolylmethyl)­amine, sodium (l)-ascorbate, t-BuOH, H_2_O, rt, overnight; (c) **7**, Et_3_N, DMSO, 40 °C, 1 h.

## Radiolabeling

Four precursors are available for radiolabeling either with ^133^La (**mcp-M-alb-PSMA** and **mcp-D-alb-PSMA**) requiring the macropa chelator and with ^123^I (**mcp-M-Sn-alb-PSMA** and **mcp-D-Sn-alb-PSMA**) containing the trimethylstannyl leaving group.

To evaluate the influence of chelator loading (the presence of the metal cation in the chelator) on the ^123^I-radiotracer pharmacological properties, radiolabeling was performed using [^123^I]­I^–^ to obtain the La-containing (**La-mcp-M-[^123^I]­alb-PSMA**) and the metal-free (**mcp-M-[^123^I]­alb-PSMA**) radiotracer. This allows a direct comparison in terms of binding affinity and pharmacokinetics. For the ^133^La labeling, the respective precursors **mcp-M-alb-PSMA** and **mcp-D-alb-PSMA** ^29^ containing nonradioactive iodine were used. By systematically assessing these variations, potential alterations in biodistribution and imaging performance could be identified, contributing to a more comprehensive understanding of the radiotracer behavior with different radionuclides and under different conditions. All albumin-binding radioconjugates studied in this work contain the albumin binder 4-(4-iodophenyl)­butyrate (herein expressed as **alb**) and are displayed in Figure 2. Additionally, previously published derivatives **mcp-M-PSMA** (monovalent) and **mcp-D-PSMA** (bivalent)^30^ without the albumin-binding unit as well as **PSMA-617** containing the DOTA chelator were used for comparison.

**Figure 2:**
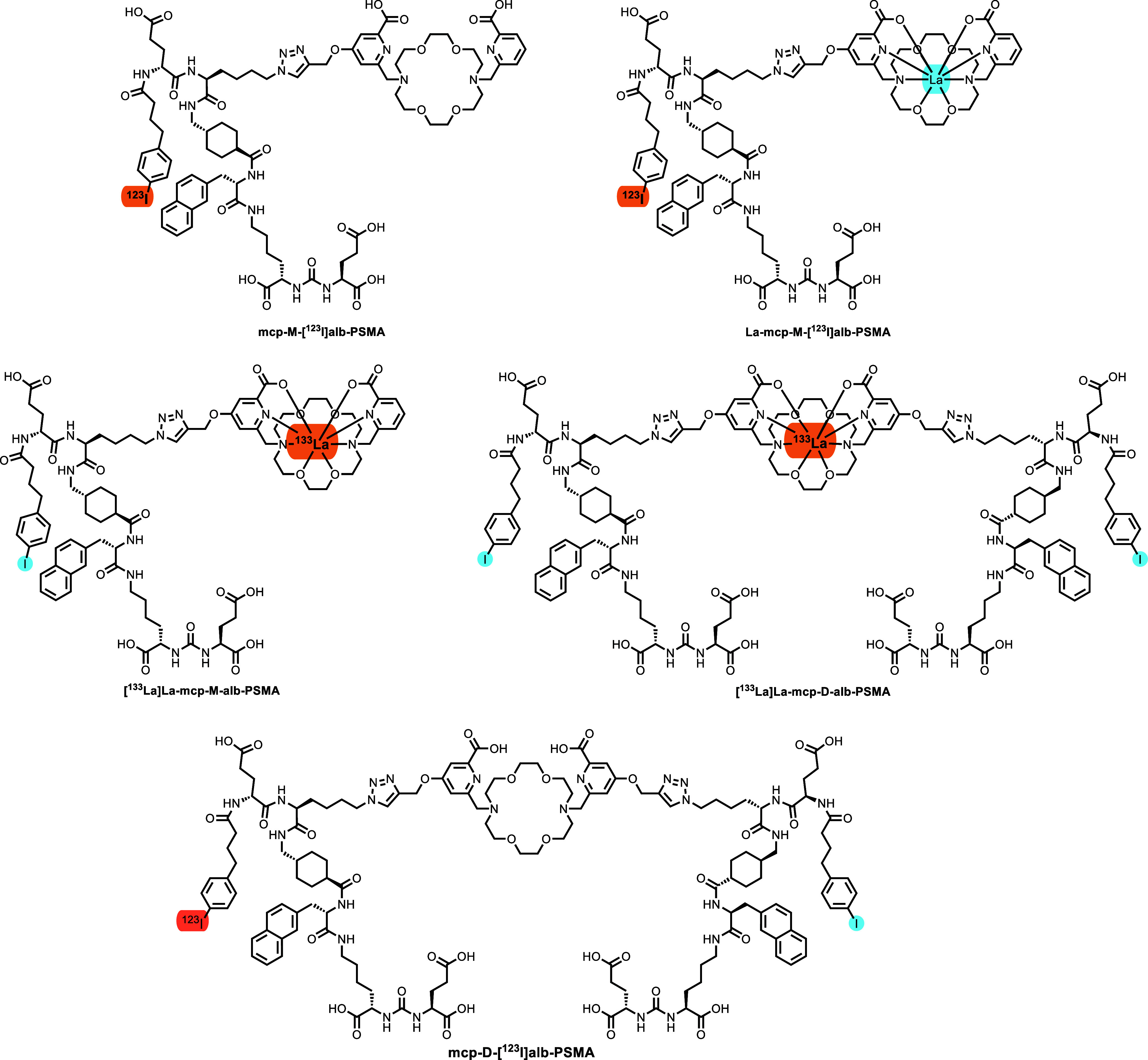
Overview of all investigated albumin-binding radioligands **mcp-M-[^123^I]­alb-PSMA**, **La-mcp-M-[^123^I]­alb-PSMA,** and **[** ^**133**^ **La]­La-mcp-M-alb-PSMA** (monovalent) and **[** ^**133**^ **La]­La-mcp-D-alb-PSMA** (bivalent). The radionuclides marked in orange indicate the position of the radionuclide, and the atoms highlighted in blue are the nonradioactive counterparts. Chemical structure of the bivalent, bispecific radiotracer **mcp-D-[^123^I]­alb-PSMA** with one albumin binder being radioiodinated with ^123^I (highlighted in orange) and one with nonradioactive iodine (highlighted in blue).

### Radiolabeling with ^123^I

To achieve the introduction of radioiodine at the aromatic moiety of the albumin binder, an electrophilic radioiodination^33^ of the monovalent, bispecific radioconjugate was carried out using reaction tubes coated with iodogen to ensure the oxidation of [^123^I]­I^–^. Using optimized labeling conditions, the tube was rinsed with water and the precursor **mcp-M-Sn-alb-PSMA** dissolved in DMSO, EtOH, and phosphate buffer (0.18 M, pH 6) was added. The buffer was used to suppress the formation of byproducts. The radiolabeling reaction was initiated by the addition of [^123^I]­I^–^ (0.02 M NaOH solution, up to 10 GBq) and allowed to proceed for 25 min at room temperature. The reaction was stopped by transferring the solution into a glass vial (for nonradioactive La-complexation, an excess of La­(NO_3_)_3_ was added at this stage to investigate the influence of chelator loading regarding its charge and molecule geometry), followed by semipreparative HPLC purification. Afterward, the product fraction was diluted with water, applied to a C18 cartridge, rinsed with water, and eluted with EtOH. The solvent was then evaporated, and the residue was taken up in 0.9% NaCl solution and analyzed by analytical HPLC. Both radioconjugates **mcp-M-[^123^I]­alb-PSMA** and **La-mcp-M-[^123^I]­alb-PSMA** were obtained in radiochemical yields of 10% (d.c.) independent of the La-complexation and with a purity exceeding 98% (Figure 3).

**Figure 3:**
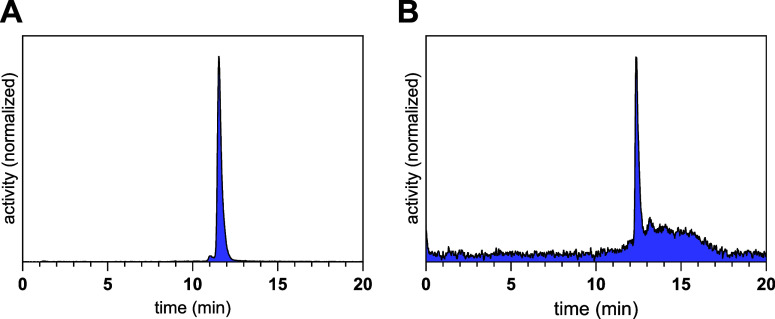
Radio-HPLC-chromatograms of the purified radioiodinated radioligands **mcp-M-[^123^I]­alb-PSMA** with t_R_ = 11.5 min (A) and **mcp-D-[^123^I]­alb-PSMA** with t_R_ = 12.2 min (B).

The procedure for the bivalent, bispecific conjugate **mcp-D-Sn-alb-PSMA** was the same with the distinct difference that nonradioactive NaI (100 nmol) was added after the radiolabeling step with [^123^I]­I^–^ to the iodination tube in order to convert both binding sites to iodine (Figure 2). Due to the fact that the biodistribution data of the previously published bivalent, bispecific **[** ^**225**^ **Ac]­Ac-mcp-D-alb-PSMA** conjugate were not convincing due to a high retention in the spleen and kidneys^29^ and that **mcp-D-[^123^I]­alb-PSMA** (Figure 2) has a low molar activity due to the addition of NaI during the labeling procedure, we decided not to further optimize the radioiodination and to exclude this radiotracer from *in vivo* experiments.

### Radiolabeling with ^133^La

It was previously shown by us and others that lanthanum-133 acts as an excellent diagnostic surrogate for actinium-225 using macropa-containing radiotracers.^24,25,27,34^ The radiolabeling process is convenient and was conducted at room temperature for 30 min in ammonium acetate buffer (pH 6.0), leading to almost quantitative radiochemical conversion with a radiochemical purity exceeding 95% for both radioconjugates **[** ^**133**^ **La]­La-mcp-M-alb-PSMA** and **[** ^**133**^ **La]­La-mcp-D-alb-PSMA** as determined by radio-TLC and radio-HPLC analyses (Figure 4).

**Figure 4:**
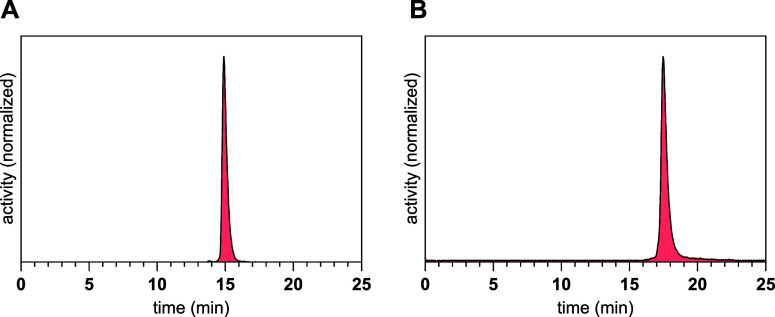
Radio-HPLC-chromatograms of **[** ^**133**^ **La]­La-mcp-M-alb-PSMA** with t_R_ = 14.9 min (A) and **[** ^**133**^ **La]­La-mcp-D-alb-PSMA** with t_R_ = 17.5 min (B); free [^133^La]­La^3+^ appears at t_R_ = 2.5 min.

In addition, the stability of **[** ^**133**^ **La]­La-mcp-M-alb-PSMA** in terms of degradation and complex dissociation was investigated at different time points (Figure 5), representative of the chemically identical radioiodinated ligand **La-mcp-M-[** ^**123**^ **I]­alb-PSMA**, which is expected to behave equally. The radioconjugate was added to human serum and incubated at 37 °C for up to 24 h, and the stability was evaluated by radio-HPLC at three distinct time points. As a result, no proteolytic degradation was observed over 24 h (Figure 5).

**Figure 5:**
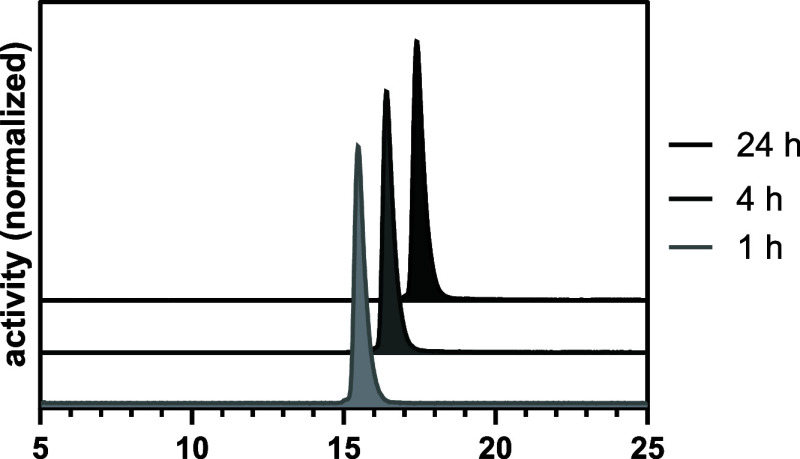
Stability determination of **[** ^**133**^ **La]­La-mcp-M-alb-PSMA** human serum after incubation at 37 °C. Samples were taken after 1, 4, and 24 h and analyzed by radio-HPLC.

## Radiopharmacological Characterization *in Vitro*

To exclude the possibility that differences in the *in vivo* behavior arise from altered target binding, the binding affinity and internalization of PSMA precursors with and without a metal chelate were first assessed. The PSMA-binding affinities of the macropa-based, albumin-binding PSMA ligands **mcp-M-alb-PSMA** and **mcp-D-alb-PSMA** were determined with or without La^3+^ complexation in a competitive binding assay using PSMA-positive LNCaP cells with **[** ^**133**^ **La]­La-PSMA-617** as a radiolabeled standard (Figure 6, Table 1; see Supporting Information in Figure S1 for details on saturation binding of **[** ^**133**^ **La]­La-PSMA-617**).

**Figure 6:**
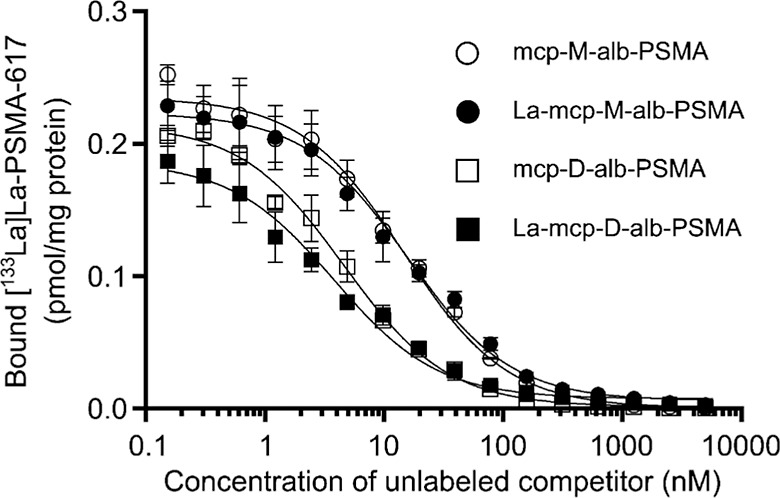
Competitive binding curves comparing the cell binding of **mcp-M-alb-PSMA** and **mcp-D-alb-PSMA** with and without La^3+^-content, respectively. Binding affinity to PSMA-expressing LNCaP cells was determined with **[** ^**133**^ **La]­La-PSMA-617** (K_d_ = 1.5 nM, c_radioligand_ = 1 nM) as the radioligand.

**Table 1:** In Vitro Binding Data of PSMA-Targeting Ligands in Their La-Containing and Metal-Free Forms Determined on PSMA-Positive LNCaP Cells With **[** ^**133**^ **La]­La-PSMA-617** (K_d_ = 1.5 nM, c_radioligand_ = 1 nM) as a Radioligand

ligand	*K*_i_ (nM)^a^
mcp-M-alb-PSMA	8.9 (7.3–10.7)^b^
La-mcp-M-alb-PSMA	9.4 (7.5–11.8)^b^
mcp-D-alb-PSMA	2.8 (2.4–3.2)^b^
La-mcp-D-alb-PSMA	2.3 (1.8–3.0)^b^

a One experiment that was performed in triplicate.

b 95% confidence interval.

As shown in Figure 7, the PSMA-targeting ligands **mcp-M-alb-PSMA** and **mcp-D-alb-PSMA** inhibited the binding of **[** ^**133**^ **La]­La-PSMA-617** to human LNCaP prostate carcinoma cells in a concentration-dependent manner. The obtained data (Table 1) indicate an improved PSMA-binding affinity of the bivalent derivative compared to the monovalent one translating into lower *K*_i_ values for **mcp-D-alb-PSMA** compared to **mcp-M-alb-PSMA**, which corresponds to the data obtained for the respective ^225^Ac-radioconjugates.^29^ However, these differences are not complexation-dependent, as similar *K*_i_ values were obtained for the individual PSMA inhibitors in the La-containing and their metal-free forms. In this case, the metal cation in the macropa moiety exerts no substantial influence on the *in vitro* behavior of the PSMA ligands, and their nanomolar affinity is independent of complexation.

**Figure 7:**
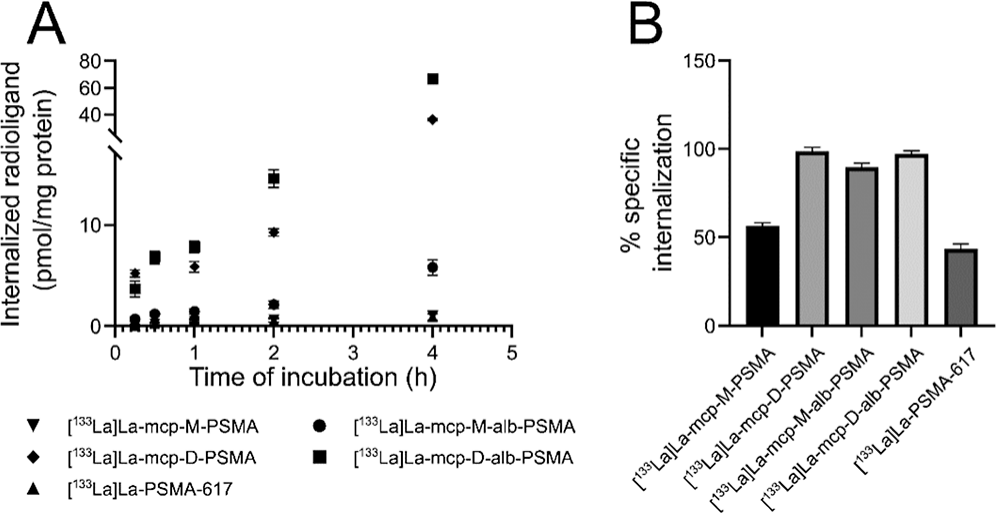
Time-dependent cellular uptake of the four macropa-based PSMA ligands in direct comparison to **[** ^**133**^ **La]­La-PSMA-617**. The extent of PSMA-specific internalization (A) was assayed over a period of 4 h by incubating the PSMA-expressing LNCaP cells with 10 nM of each ^133^La-labeled ligand at 37 °C. The percentage of specific internalization after incubation at 37 °C for 60 min (B) was determined in the presence of 500 μM unlabeled **PSMA-617**.

As the internalization of radioligands is generally an important parameter, especially for therapy approaches, the two albumin-binding PSMA radioligands **[** ^**133**^ **La]­La-mcp-M-alb-PSMA** and **[** ^**133**^ **La]­La-mcp-D-alb-PSMA** were further analyzed in terms of cell uptake and internalization in comparison to their previously published counterparts without albumin-binding moieties **[** ^**133**^ **La]­La-mcp-M-PSMA** and **[** ^**133**^ **La]­La-mcp-D-PSMA** as well as **[** ^**133**^ **La]­La-PSMA-617**. All five radioligands were incubated with PSMA-positive LNCaP cells for a period of up to 4 h at 37 °C, and the specific internalization was evaluated as a function of time (Figure 8, Table 2).

**Figure 8:**
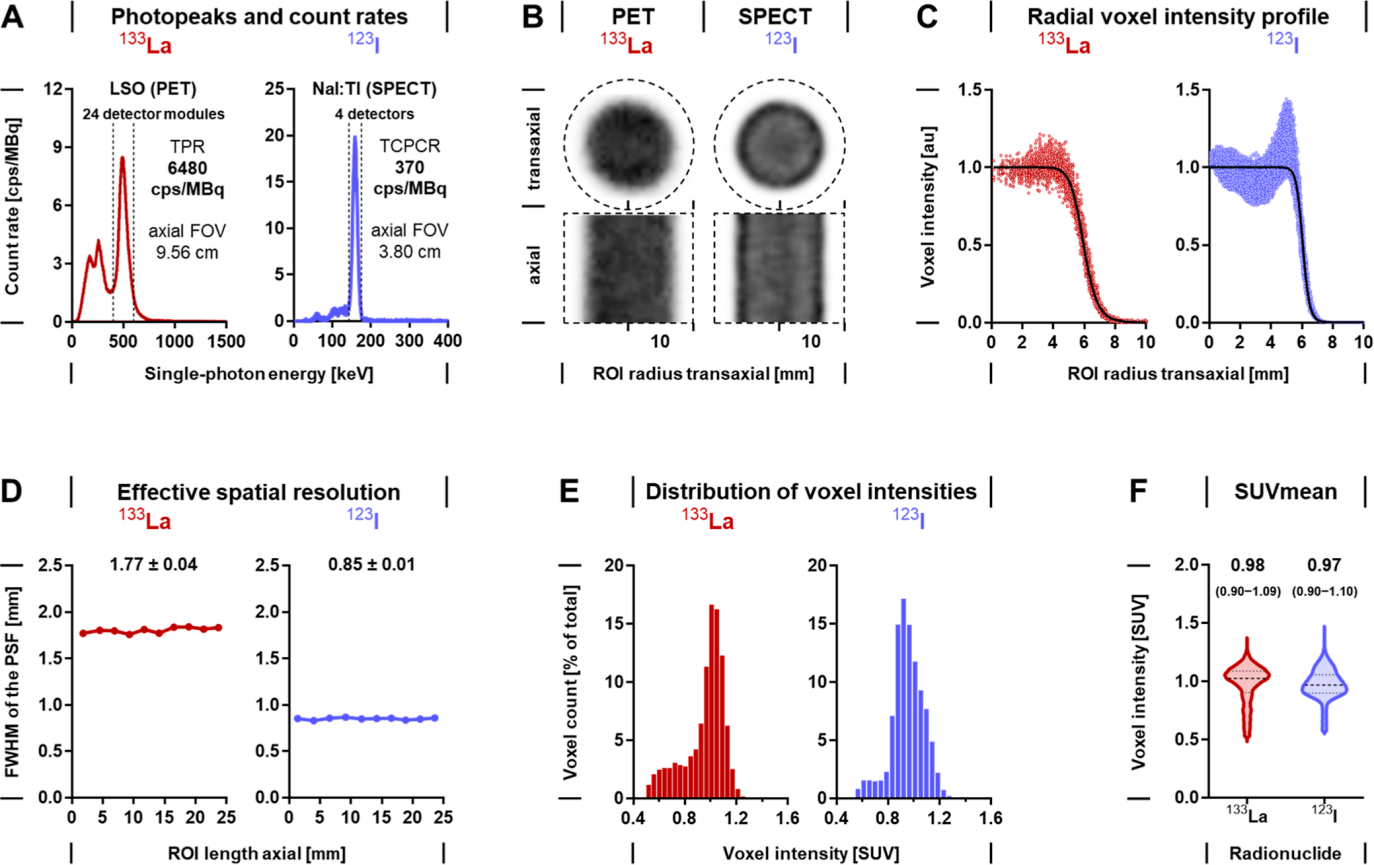
Sensitivity, spatial resolution, and quantification accuracy in PET imaging of ^133^La and SPECT imaging of ^123^I in small-animal imaging systems; 5 mL syringe activity phantoms containing an activity concentration of 7.5 MBq/mL were measured using the nanoScan PET/CT and the nanoScan SPECT/CT equipped with the APT56 aperture consisting of four multipinhole ultrahigh-energy collimators; (A) energy spectra of photons with 20% energy windows of the recorded photopeaks and count rates for imaging of ^133^La (511 keV) and ^123^I (159 keV); detector materials: LSO, lutetium oxyorthosilicate crystals, NaI:Tl, thallium-doped sodium iodide scintillators; TPR, total prompt rate; TCPCR, total collimated photon count rate; FOV, field-of-view; (B) planar SPECT images of activity phantoms; dashed fields indicate ROIs used for image analysis; (C) radial voxel intensity profiles showing the decrease in contrast at the edges of the activity phantoms along the transaxial radius of the ROI; (D) effective spatial resolution along the axial length of the analyzed ROI; the spatial resolution was determined as full width at half-maximum (fwhm) of the point spread function (PSF), means ± standard error; (E) distribution of voxel intensities within the activity phantoms; the analyzed ROI included all voxels above the minimum intensity threshold of 39%; (F) statistics of the mean standardized uptake value (SUV_mean_) measured in the activity phantoms; median with 25th and 75th percentiles.

**Table 2:** In Vitro Internalization Data of PSMA-Targeting Ligands Obtained with PSMA-Positive LNCaP Cells upon Incubation with 10 nM of Each ^133^La-Labeled Ligand at 37 °C for 60 min

ligand	specific cell surface-binding (pmol/mg protein)^a^	specific internalization (pmol/mg protein)^a^	specific internalization (%)^a^
[^133^La]La-mcp-M-PSMA	0.86 ± 0.10	0.49 ± 0.10	56.4
[^133^La]La-mcp-M-alb-PSMA	1.61 ± 0.41	1.45 ± 0.27	89.7
[^133^La]La-mcp-D-PSMA	5.96 ± 0.42	5.88 ± 0.53	98.7
[^133^La]La-mcp-D-alb-PSMA	8.05 ± 0.54	7.83 ± 0.58	97.2
[^133^La]La-PSMA-617	1.13 ± 0.39	0.49 ± 0.23	43.4

a One experiment that was performed in triplicate.

A time-dependent cell uptake of varying extent was observed for the five investigated radioligands, whereby the radioligands with two PSMA-binding motifs **[** ^**133**^ **La]­La-mcp-D-PSMA** and **[** ^**133**^ **La]­La-mcp-D-alb-PSMA** possess a substantially higher specific internalization compared to the monovalent radioligands **[** ^**133**^ **La]­La-mcp-M-PSMA**, **[** ^**133**^ **La]­La-mcp-M-alb-PSMA,** and the standard **[** ^**133**^ **La]­La-PSMA-617** (Figure 7A). Of the three monovalent radioligands, **[** ^**133**^ **La]­La-mcp-M-alb-PSMA** is preferentially internalized by LNCaP cells compared to the other two lacking an albumin binder. A closer look at the time course of relative internalization reveals that for the bivalent radioligands **[** ^**133**^ **La]­La-mcp-D-PSMA** and **[** ^**133**^ **La]­La-mcp-D-alb-PSMA**, more than 97% of the PSMA-specific cell-associated tracer amount is found intracellularly already after 60 min (Figure 7B, Table 2). At the same time, this value approximates 90% for **[** ^**133**^ **La]­La-mcp-M-alb-PSMA**, while it is 56% and 43% for **[** ^**133**^ **La]­La-mcp-M-PSMA** and **[** ^**133**^ **La]­La-PSMA-617**, respectively. It can be concluded that the absolute amount of radioligand incorporated by the cells is higher for the bivalent radioligands than that for their monovalent counterparts. These *in vitro* results demonstrate that PSMA affinity and internalization are unaffected by chelator loading. Radioiodinated ligands without an additional complexed metal have the same PSMA-binding affinity.

## Lanthanum-133 versus Iodine-123 in Quantitative PET and SPECT Imaging

As a prerequisite, PET images of ^133^La- and SPECT images of ^123^I-loaded activity phantoms were analyzed to evaluate radionuclide-specific sensitivity, effective spatial resolution, and accuracy in image-based quantification achieved with preclinical imaging systems. Owing to the emission of positrons during nuclear transformation of ^133^La, the measurement of gamma-photon coincidences of (511 keV ±20%) provided a total prompt rate of 6480 cps/MBq detected simultaneously within the 9.56 cm axial field-of-view of the Mediso nanoScan PET/CT (Figure 8A). In comparison, measuring the single-photon emission of ^123^I (159 keV ±20%) provided a lower total photon count rate of 370 cps/MBq detected within the 3.8 cm axial field-of-view of the Mediso nanoScan SPECT/CT equipped with ultrahigh-energy collimators to prevent quantification errors from backscattering of high-energy photons (590 keV). Due to its higher count rate, PET imaging of ^133^La provided a time resolution in the range of seconds, whereas SPECT imaging of ^123^I required scan times in the range of minutes to hours due to the necessity for stepwise recording of projections at different angles and bed positions. On the other hand, ^123^I provided favorable count rates enabling quantitative SPECT imaging at time points later than 24 h after injection due to its longer physical half-life compared to that of ^133^La.

The effective spatial resolution was considerably higher in SPECT images of ^123^I (0.85 mm) compared to PET images of ^133^La (1.77 mm) (Figure 8B–D). The voxels from the syringe activity phantom contents showed a similar intensity distribution for both ^133^La and ^123^I, indicating a comparable accuracy in image quantification for both imaging modalities, allowing a direct comparison of PET and SPECT data (Figure 8E–F).

## Biodistribution Using LNCaP Tumor-Bearing Mice

The locoregional distribution of the five ^133^La-labeled **[** ^**133**^ **La]­La-PSMA-617**, **[** ^**133**^ **La]­La-mcp-M-PSMA, [** ^**133**^ **La]­La-mcp-D-PSMA**, **[** ^**133**^ **La]­La-mcp-M-alb-PSMA**, and **[** ^**133**^ **La]­La-mcp-D-alb-PSMA** as well as the two ^123^I-labeled PSMA-targeting radioligands **La-mcp-M-[** ^**123**^ **I]­alb-PSMA** and **mcp-M-[** ^**123**^ **I]­alb-PSMA** was visualized via small-animal PET and SPECT analyses using LNCaP tumor-bearing mice until 22 and 44 h, respectively, after intravenous injection (Figure 9 A). Quantitative image analysis provided time courses of region-averaged standardized uptake values (SUV_mean_) in the blood content of the heart, kidneys, liver, lung, muscle, LNCaP tumors, parotid glands, thyroid gland, and urinary bladder (Figure 9 B–C).

**Figure 9:**
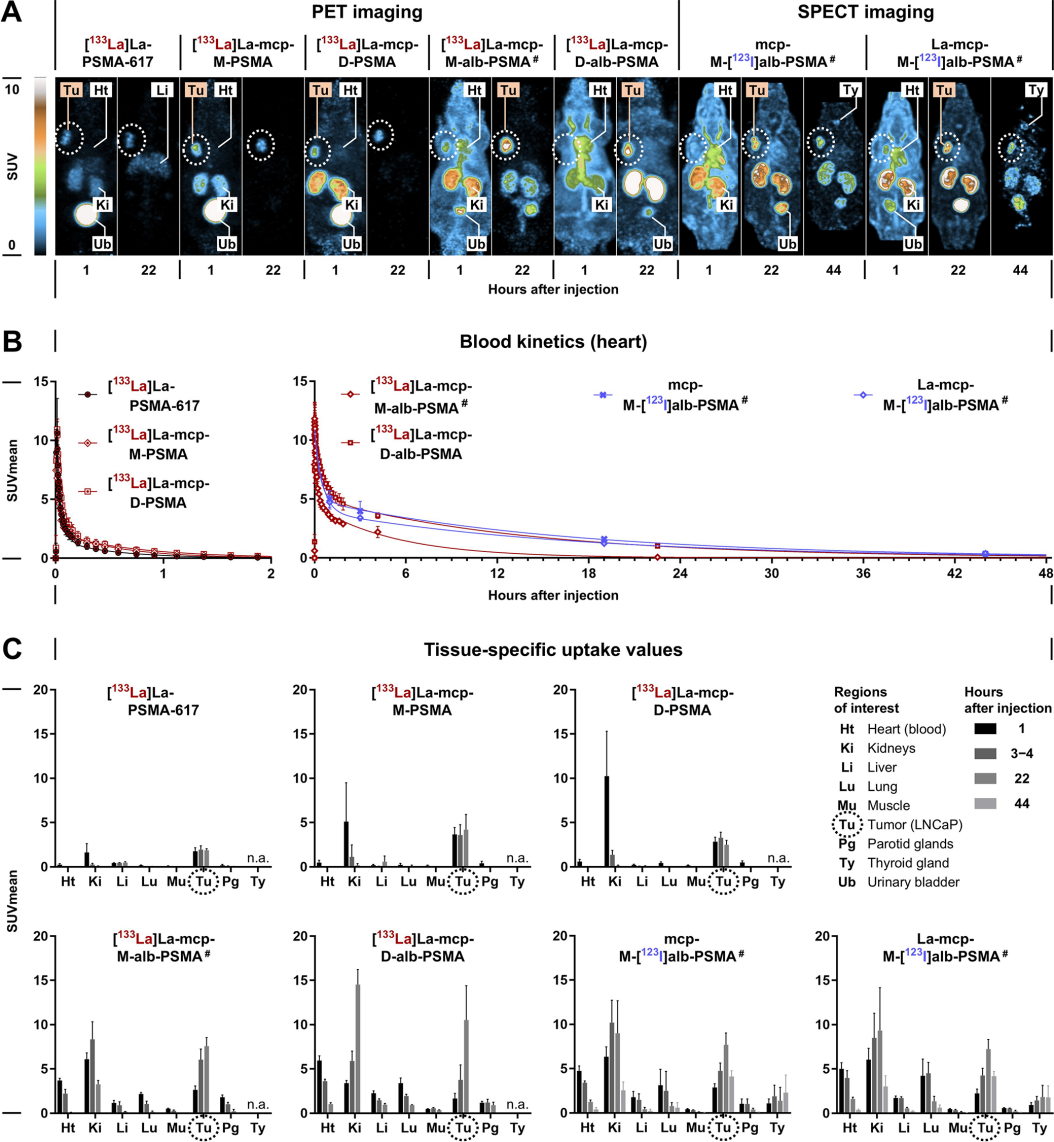
Biodistribution of ^133^La- and ^123^I-labeled PSMA radioligands in LNCaP tumor-bearing mice determined by quantitative PET and SPECT imaging analysis, respectively; (**A**) maximum-intensity projections of radioligand uptake at indicated time points after injection and common scale; (**B**) time-resolved changes in uptake values in the blood content of the heart; data fitted with the “two-phase decay” nonlinear regression model; blood half-lives are provided in Table 3; (**C**) region-averaged uptake values in specific tissues at indicated time points after radioligand injection; data presented as mean values with standard deviation; numbers of replicates and experiments are provided in Table 3; (SUV) standardized uptake value; ^#^ indicates corresponding radiohybrid ligands.

Blood kinetics: all seven PSMA radioligands showed biphasic blood kinetics with distinct biological half-lives for their distribution and elimination (Table 3). The reference PET radiotracer **[** ^**133**^ **La]­La-PSMA-617**, the monovalent **[** ^**133**^ **La]­La-mcp-M-PSMA**, and the bivalent **[** ^**133**^ **La]­La-mcp-D-PSMA** showed similar blood elimination half-lives shorter than 30 min. The monovalent, bispecific **[** ^**133**^ **La]­La-mcp-M-alb-PSMA** and the bivalent, bispecific **[** ^**133**^ **La]­La-mcp-D-alb-PSMA** showed prolonged blood retention, with half-lives in the range of hours, which increased with the number of albumin binders per radioligand.

**Table 3:** PET and SPECT Image-Derived Parameters Describing the Pharmacokinetic Profiles of ^133^La- and ^123^I-Labeled PSMA-Radioligands in the Blood and Total Body Mass of LNCaP Tumor-Bearing Mice

radioligand	blood^a^			total body^b^	
	distribution		elimination		
	fraction [ %]	half-life [min]	half-life [h]	half-life [h]	*n* (exp.^c^)
**[** ^**133**^ **La]La-PSMA-617**	85.8 (79.0–92.6)***	2.10 (1.57–2.62)	0.37 (0.30–0.45)	0.45 (0.35–0.56)	4 (2,2)
**[** ^**133**^ **La]La-mcp-M-PSMA**	79.0 (58.1–100)***	1.35 (0.93–2.42)	0.40 (0.35–0.45)	0.84 (0.49–1.20)	2 (2)
**[** ^**133**^ **La]La-mcp-D-PSMA**	82.3 (79.0–85.5)***	1.78 (1.20–2.35)	0.46 (0.25–0.67)	0.94 (0.90–0.98)	4 (2,2)
**[** ^**133**^ **La]La-mcp-M-alb-PSMA** ^*#*^ ^d^	63.0 (58.3–67.7)	5.11 (3.26–6.96)	3.15 (2.07–4.24)	7.87 (7.32–8.43)	6 (2,2,2)
**[** ^**133**^ **La]La-mcp-D-alb-PSMA**	53.0 (48.5–57.5)**	21.4 (15.6–27.2)***	9.54 (8.42–10.7)***	106 (73.5–139)***	4 (2,2)
**mcp-M-[** ^**123**^ **I]alb-PSMA** ^#^	53.2 (31.2–75.2)*	17.1 (12.3–21.8)*	12.3 (7.59–17.0)***	16.0 (10.6–21.4)	5 (3,2)
**La-mcp-M-[** ^**123**^ **I]alb-PSMA** ^#^	64.9 (62.8–67.1)	20.3 (14.8–25.9)***	12.0 (10.5–13.6)***	14.2 (10.4–18.1)	9 (2,3,4)

a Time-activity courses were analyzed by the nonlinear regression models for a two-phase exponential decay and.

b One-phase exponential decay (see experimental section for details); *n* represents the number of replicates (animals) investigated in total and.

c Per independent experiment (exp.); data presented as means (with 95% confidence interval).

d Significance of differences compared to **[** ^**133**^ **La]­La-mcp-M-alb-PSMA**: *p < 0.05, **p < 0.01, ***p < 0.001; ^#^ indicates corresponding radiohybrid ligands

Unexpectedly, the blood elimination half-lives of the monovalent, bispecific SPECT radiotracers **mcp-M-[** ^**123**^ **I]­alb-PSMA** and **La-mcp-M-[** ^**123**^ **I]­alb-PSMA** with 12.3 and 12.0 h, respectively, were significantly longer compared to their corresponding PET radiotracer **[** ^**133**^ **La]­La-mcp-M-alb-PSMA** with 3.15 h. Of note, both the La-free **mcp-M-[** ^**123**^ **I]­alb-PSMA** and the La-containing conjugate **La-mcp-M-[** ^**123**^ **I]­alb-PSMA** exhibited similar kinetic profiles in the blood, indicating that the presence of La^3+^ in the macropa chelator has no influence on the blood half-lives of the radiohybrid ligands. Results of additional experiments also showed that different radiolabeling matrices and procedures as well as differences in the amounts of substance administered (molar activity) cannot explain this unexpected phenomenon (Supporting Information, Figure S3A,B).

The difference in image-extracted blood kinetics between monovalent, bispecific SPECT radiotracer **La-mcp-M-[** ^**123**^ **I]­alb-PSMA** and chemically identical PET radiotracer **[** ^**133**^ **La]­La-mcp-M-alb-PSMA** was successfully validated by the analysis of blood samples (Figure 10 A). The uptake values extracted from images and those determined in the corresponding blood samples showed significant linear-positive relationships (Figure 10 B). These results demonstrate that quantitative PET and SPECT studies in mice provide reliable quantitative data for the preclinical pharmacokinetic evaluation of ^133^La- and ^123^I-labeled radiotracers *in vivo*.

**Figure 10:**
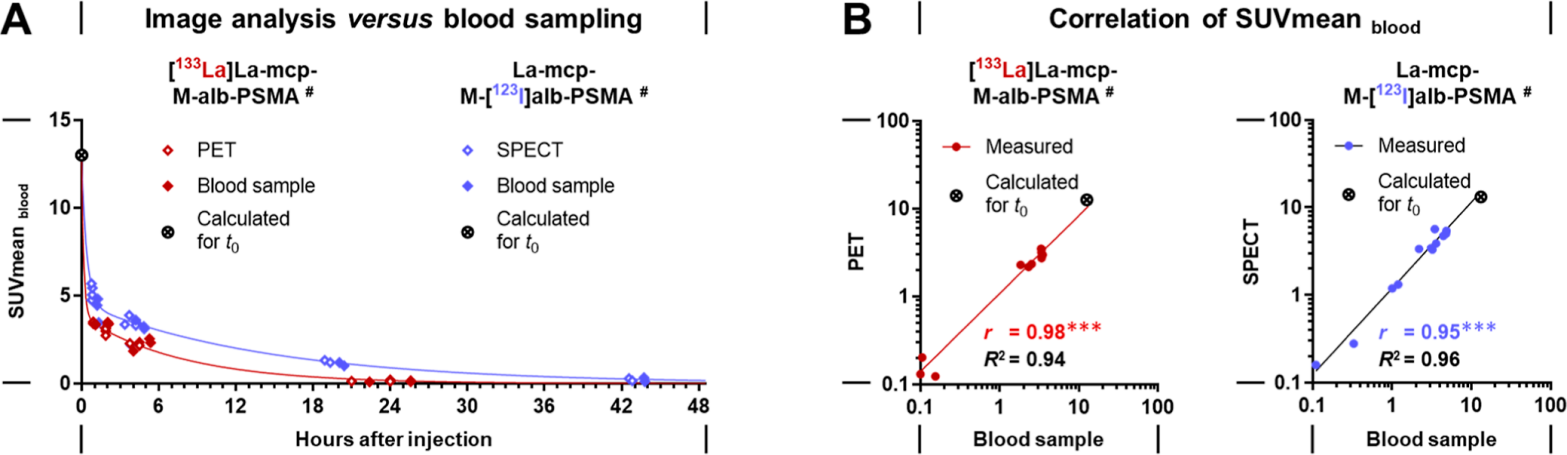
Image- and sample-derived uptake values of ^133^La- and ^123^I-labeled PSMA radioligands in the blood; (A) radioligand kinetics in blood; PET and SPECT image-extracted values from analyzing the blood content of the heart; corresponding blood samples were collected within the following time periods after radioligand injection: 1–2 h and 3–5 h (retrobulbar sampling, *n* = 4), followed by either 20–26 h or 43–44 h (terminal sampling by cardiac puncture, *n* = 2); (B) linear relationships between sample-derived and image-extracted uptake values; (*t*_0_) time point of radioligand injection; theoretical initial uptake values in mouse blood were calculated based on published data for total blood volume per body weight (see methods section for details); (*r*) Pearson’s correlation coefficient with significance of linear relationships: ***p < 0.001; (*R* ^2^) goodness of curve fit using the linear regression model.

Tumor uptake: all ^133^La- and ^123^I-labeled PSMA-targeting radioligands showed specific uptake in subcutaneous LNCaP tumor xenografts in mice, enabling their visual detection in PET and SPECT images, respectively. The reference PET radiotracer **[** ^**133**^ **La]­La-PSMA-617** showed the lowest uptake values in tumors reaching the maximum 1 h p.i. Within the same time, the monovalent **[** ^**133**^ **La]­La-mcp-M-PSMA** and the bivalent **[** ^**133**^ **La]­La-mcp-D-PSMA** reached 1.6–2.2-fold higher uptake values. The highly stable retention of the three ^133^La-labeled radioligands in the LNCaP tumors for at least 22 h is comparable to the reported therapeutic radioligand **[** ^**177**^ **Lu]­Lu-PSMA-617** in the same model^35^ and the results for the respective ^225^Ac-radioligands published recently.^30^ Since **[** ^**177**^ **Lu]­Lu-PSMA-617** is also well known for undergoing receptor-mediated endocytosis in LNCaP-tumor cells after specific binding to PSMA,^35^ the stable tumor retention of all three ^133^La-labeled radioligands may result from similar uptake fractions to be efficiently trapped in the tumor cells.

The albumin and PSMA-binding monovalent, bispecific **[** ^**133**^ **La]­La-mcp-M-alb-PSMA** as well as the bivalent, bispecific **[** ^**133**^ **La]­La-mcp-D-alb-PSMA** showed 1.8-fold and 3.2-fold higher uptake in LNCaP tumors compared to their respective albumin binder-free counterparts. The tumor accumulation of **[** ^**133**^ **La]­La-mcp-M-alb-PSMA** occurred faster compared to that of **[** ^**133**^ **La]­La-mcp-D-alb-PSMA**, depending either on the different molar mass of the radioconjugates and/or the availability of the free (nonalbumin-bound) radioligand fraction in the extravascular space, which is expected to decrease with the higher number of albumin-binding entities. The same behavior was also observed for the respective ^225^Ac-labeled radioconjugates.^29^

Both monovalent, bispecific SPECT radiotracers **mcp-M-[** ^**123**^ **I]­alb-PSMA** (SUV_mean_ = 7.2 ± 1.1) and **La-mcp-M-[** ^**123**^ **I]­alb-PSMA** (SUV_mean_ = 7.7 ± 1.3) exhibit a similar tumor uptake compared to their corresponding PET radiotracer **[** ^**133**^ **La]­La-mcp-M-alb-PSMA** (SUV_mean_ = 7.6 ± 1.0). The results demonstrate a successful application of the radiohybrid concept regarding tumor uptake. Of advantage, the physical half-life of ^123^I enables an extended monitoring of the SPECT radiotracer pharmacokinetics compared to ^133^La-labeled PET radiotracers, showing a 50% decrease in the uptake values of **mcp-M-[** ^**123**^ **I]­alb-PSMA** and **La-mcp-M-[** ^**123**^ **I]­alb-PSMA** in tumors between 22 and 44 h. Of note, the respective ^225^Ac-derivatives show a different *in vivo* behavior, probably due to their different molar activity.^29^ Of note, the higher blood retention of these both monovalent, bispecific SPECT radiotracers compared to their corresponding PET radiotracer **[** ^**133**^ **La]­La-mcp-M-PSMA** has no effect on the uptake in tumors.

Excretion: both the ^133^La- and the ^123^I-labeled PSMA-targeting radioligands were excreted via the renal pathway as the reference PET radiotracer **[** ^**133**^ **La]­La-PSMA-617** showing the most rapid elimination from the body, despite some residual off-target accumulation of free [^133^La]­La^3+^ in the liver (Table 3, Figure 9C), which is the major drawback of using DOTA in combination with ^225^Ac and ^133^La. In contrast, the macropa-conjugated derivatives showed no evidence of sustained nonspecific liver accumulation, as the hepatic activity declined over time. In comparison, the slower excretion of monovalent **[** ^**133**^ **La]­La-mcp-M-PSMA** and bivalent **[** ^**133**^ **La]­La-mcp-D-PSMA** occurred mainly due to their higher retention in the kidneys, which increased with the number of PSMA-binding entities. The further slowdown in excretion observed for the monovalent, bispecific **[** ^**133**^ **La]­La-mcp-M-alb-PSMA** and the bivalent, bispecific **[** ^**133**^ **La]­La-mcp-D-alb-PSMA** resulted from their higher retention in both blood and kidneys and increased with the number of albumin-/PSMA-binding entities per radioligand. This behavior in excretion has also been observed and is in good agreement for the ^225^Ac-radioconjugates with and without the albumin binder.^29,30^

The excretion of the monovalent, bispecific SPECT radiotracers **mcp-M-[** ^**123**^ **I]­alb-PSMA** and **La-mcp-M-[** ^**123**^ **I]­alb-PSMA** tended to be slower compared to their corresponding PET radiotracer **[** ^**133**^ **La]­La-mcp-M-alb-PSMA**. This is consistent with the half-lives of the radiotracers in the blood. However, the slower elimination of **mcp-M-[** ^**123**^ **I]­alb-PSMA** and **La-mcp-M-[** ^**123**^ **I]­alb-PSMA** from the renal cortex also contributed substantially to this trend.

## Stability in LNCaP Tumor-Bearing Mice

The stability of metal-based radiotracers is correlated with the stability of the formed radiometal complexes within the radioconjugate. This is of importance, especially for radioconjugates used in targeted radionuclide therapy. ^133^La with a similar coordination chemistry allows a pertinent prognosis for ^225^Ac.^28^ As shown in Figure 9A, the reference PET radiotracer **[** ^**133**^ **La]­La-PSMA-617** exhibited some residual off-target accumulation in the liver (3.5 ± 0.84% of the initially injected dose after 22 h), suggesting that the [^133^La]­La-DOTA-moiety is susceptible to enzymatic transchelation of [^133^La]­La^3+^. In contrast, no liver accumulation occurred using the PSMA-ligands containing macropa as chelator, but a prolonged retention in the blood.^25–27^ The formed [^133^La]­La-mcp-moiety shows a higher kinetic inertness, which is superior over the [^133^La]­La-DOTA-moiety. The predominant accumulation of [^133^La]­La^3+^ in the liver has already been visualized in mice via PET imaging, showing strong uptake of free [^132/135^La]­La^3+^ after intravenous injection due to its heavy metal character.^36,37^

To address the differences in blood kinetics, an *ex vivo* radiometabolite analysis was performed with both the radioiodinated and the ^133^La-labeled **mcp-M-alb-PSMA** conjugate after 4 h, showing that **[** ^**133**^ **La]­La-mcp-M-alb-PSMA** (t_R_ = 14.5 min) remained intact in blood. In both kidney homogenates and urine samples, 17–23% have been converted into a more hydrophilic radiometabolite (t_R_ = 13.8 min) detectable (Figure 11B). Compared to the radio-HPLC chromatograms of a reference compound containing 4-phenyl butyrate (without iodine) instead of 4-(4-iodophenyl)­butyrate as albumin binder, the retention time corresponds to the deiodinated version of the radioligand (t_R_ = 13.8 min, Supporting Information, Figure S6), suggesting that **[** ^**133**^ **La]­La-mcp-M-alb-PSMA** is susceptible to enzymatic conversion by kidney deiodinases catalyzing the release of iodide from the iodophenyl group of the albumin-binding entity. Both type-1 and type-2 deiodinases are known to be present and active in medullary and cortical kidney tissues.^38^ Deiodination in the kidneys is also supported by the tissue-specific radio-HPLC profiles of radiometabolites derived from the SPECT radiotracer **La-mcp-M-[** ^**123**^ **I]­alb-PSMA** *in vivo*, showing an additional peak at t_R_ = 3.6 min with 22% exclusively in urine samples 4 h p.i. (Figure 11A). Its retention time corresponded to that of free [^123^I]­I^–^ (t_R_ = 3.6 min). These results indicate that deiodination of both **[** ^**133**^ **La]­La-mcp-M-alb-PSMA** and **La-mcp-M-[** ^**123**^ **I]­alb-PSMA** occurs only during the excretion process and therefore plays no role in the different blood retention of the chemically identical radioligands. Furthermore, the absence of free [^123^I]­I^–^ in blood and the very low amounts taken up by the thyroid gland (<0.5% of the initially injected dose after 44 h p.i.) support the assumption that both radioligands are largely stable against deiodination in the blood, which is important for future radiotherapeutic applications and the later translation into the clinics. The radio-HPLC chromatograms further indicate that the radiohybrid ligands are retained in blood as fully intact albumin binder conjugates. Both radioconjugates remain chemically intact during circulation, with deiodination only occurring during renal clearance, as shown in Figure 11. The absence of detectable metabolites in the blood, as confirmed by radio-HPLC analyses, shows that differences in blood retention cannot be attributed to a metabolic instability. A schematic representation of the metabolite analysis is shown in Figure 12.

**Figure 11:**
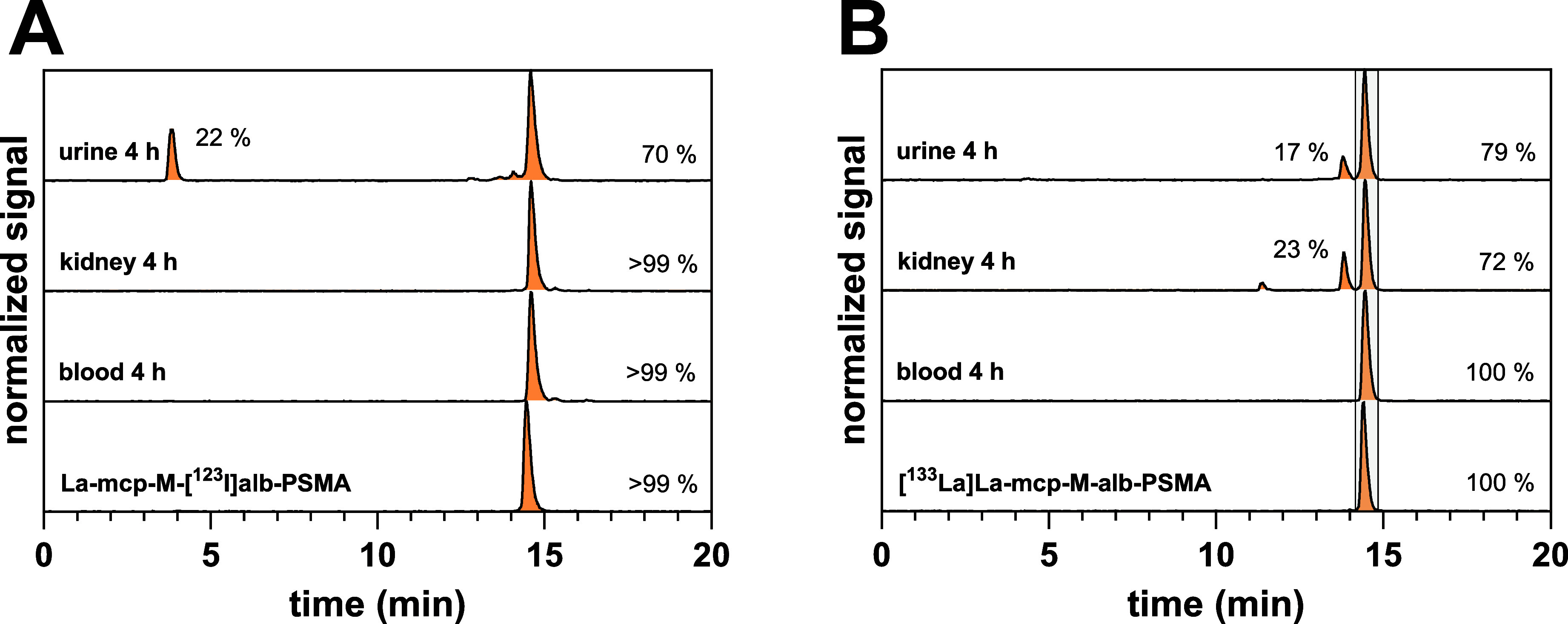
Radio-HPLC-chromatograms of **La-mcp-M-[** ^**123**^ **I]­alb-PSMA** (A) and **[** ^**133**^ **La]­La-mcp-M-alb-PSMA** (B) and of samples taken from urine, kidney, and blood at 4 h after i.v. injection of both ligands in NMRI-nu/nu mice. The percentages shown on the right refer to only the intact compound.

**Figure 12:**
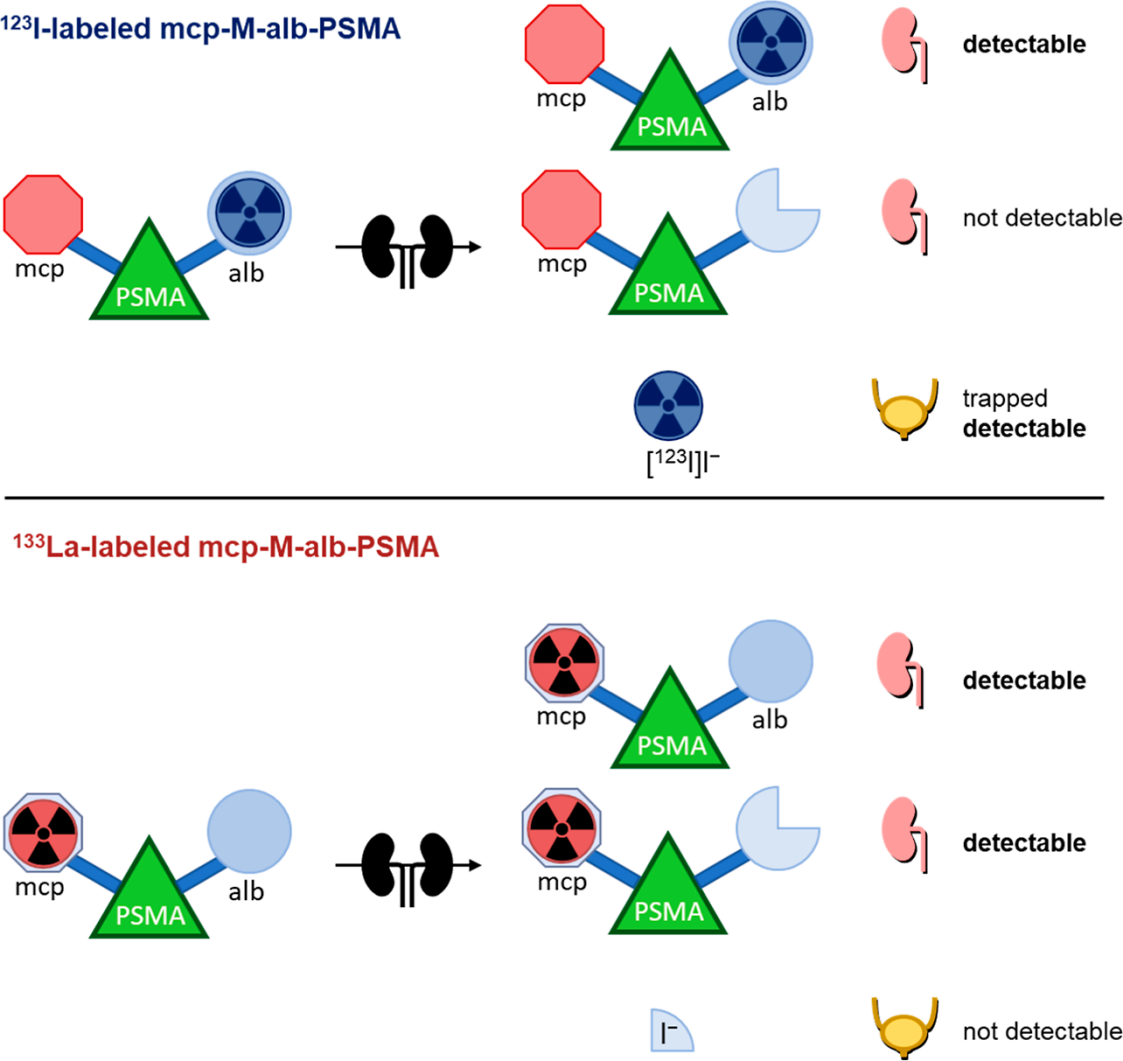
Schematic representation of the analyzed metabolites within the radiohybrid concept of the ^123^I- and ^133^La-labeled radioconjugates (mcp: macropa; alb: albumin binder; PSMA: binding motif).

## Conclusion

The novel and innovative radiohybrid approach using the radionuclide combination of ^225^Ac and ^123^I together with ^133^La as a diagnostic PET radionuclide was successfully elaborated and applied to macropa-based PSMA conjugates to enlarge the theranostic concept. This study demonstrates that radioiodine- and radiolanthanum-labeled PSMA-targeting ligands, which are based on the same chemical structure and composition containing the same binding motif, behave almost identically *in vitro* and *in vivo* with respect to PSMA-binding affinity, tumor uptake, metabolic stability, and excretion. A notable finding was observed in the significantly prolonged blood circulation time of the ^123^I-labeled conjugates in comparison to the ^133^La-labeled counterpart, while tumor accumulation remained high and unaffected. No suitable explanation for this observation could be found. Various tools such as metabolite analysis and device-specific measurement characteristics were used to clarify this phenomenon: Influences of the molar activity of the radioligands or the matrix of radiolabeling was also investigated.

Furthermore, the blood activity concentrations obtained via quantitative PET and SPECT imaging analyses were consistent with those obtained by *ex vivo* blood counting. This suggests that invasive blood sampling in animals can be avoided in future pharmacokinetic validations.

These findings highlight that even chemically identical tracers within the radiohybrid concept could exhibit distinct pharmacokinetics solely based on the position of the radionuclide. Therefore, a careful analysis of both labeling strategies is essential since a dosimetry assessment based on the radioiodine-labeled analogue may overestimate the true circulation time of corresponding radiometal-based therapeutics. This underscores the importance of performing a systematic investigation when applying the radiohybrid concept to ensure an accurate translation into clinical practice.

## Experimental Section

All chemicals were purchased from commercial suppliers and used without further purification. NMR measurements were carried out using an Agilent DD2-400 MHz NMR or Agilent DD2-600 MHz NMR spectrometer with ProbeOne. All chemical shifts of ^1^H and ^13^C signals were reported in parts per million using TMS as an internal standard at 25 °C. All spectra were calibrated using the respective solvent signal. High-resolution mass spectra (HRMS) were obtained on a Revident Q-TOF LC/Q-TOF G6575A MS (Agilent Technologies, Waldbronn, Germany) using electrospray ionization with Agilent Masshunter Workstation 3.6 software. Unless otherwise stated, the measurements were performed in bypass mode using an eluent consisting of (A) acetonitrile and (B) 0.1% formic acid in H_2_O; flow rate 0.2 mL/min. A reference mass solution containing hexakis­(1*H*,1*H*,3*H*-tetrafluoropropoxy)­phosphazene and purine was continuously coinjected via a dual AJS ESI source. Mass spectra (MALDI-MS) were recorded on a Bruker Autoflex Max MALDI/TOF-MS/MS system (Bruker, Bremen, Germany). TLC analyses for reaction control were performed on Merck Silica Gel 60 F254 TLC plates and visualized using a 254 nm UV light. Analytical HPLC was performed on a VWR Hitachi using analytical Zorbax 300SB-C18 column, 100 × 4.6 mm (Agilent Technologies, Waldbronn, Germany) and acetonitrile/water (0.1% TFA each) as mobile phase using a flow rate of 1 mL/min. Chromatographic separations were performed using automated flash column chromatography on Isolera Four (Biotage, Uppsala, Sweden) using silica gel cartridges (SNAP HC-Sfär; 5, 10, or 25 g) and reversed-phase HPLC system Knauer Azura (Knauer, Berlin, Germany) with a Zorbax 300SB-C18 semipreparative column (Agilent Technologies, Waldbronn, Germany) and acetonitrile/water (+0.1% TFA each) as mobile phase using a flow rate of 6 mL/min. **mcp-M-click**, **mcp-D-click**, **mcp-M/D-PSMA**, **mcp-M/D-alb-PSMA,** and the ^t^Bu-protected compound **4** were synthesized in accordance to the literature.^29,30^ The synthesis of the trimethylstannyl compounds **1–3** can be found in SI. Compounds **10** and **12** were not purified after the final synthesis step; instead, purification was performed following the radioiodination yielding radiochemical purities exceeding 95% as determined by radio-HPLC analysis.

### Synthesis of Compound 9

Sodium ascorbate (22 mg, 0.11 mmol, 1.1 equiv), CuSO_4_·5 H_2_O (100 mM in H_2_O, 550 μL, 1.1 equiv), and THPTA (100 mM in H_2_O, 50 μL, 0.1 equiv) were dissolved in a solution of ^t^BuOH and deionized H_2_O (0.5 mL, *v/v* = 2/1) and stirred at rt for 10 min. Compound **4** (94 mg, 0.1 mmol, 1.0 equiv) and **mcp-M-click** (59 mg, 0.1 mmol, 1.0 equiv) dissolved in ^t^BuOH and deionized H_2_O (0.5 mL, v/v = 2/1) were added, and the reaction mixture was stirred at rt overnight. Excess copper was removed by CuS-precipitation with Na_2_S (10 mg) and filtration. The solvent was removed under reduced pressure, and the residue was purified by reverse-phase column chromatography (H_2_O/acetonitrile +0.1% TFA; 90:10 → 45:55) to obtain compound **5** (44 mg, 29%) as a yellow oil after lyophilization. HRMS (ESI+): *m*/*z* = calcd 1586.6348 [M + Cu–H]^+^, found, 1586.6351.

### Synthesis of Compound 11

Sodium ascorbate (10 mg, 0.05 mmol, 1.1 equiv), CuSO_4_·5 H_2_O (100 mM in H_2_O, 500 μL, 1.1 equiv), and THPTA (100 mM in H_2_O, 50 μL, 0.1 equiv) were dissolved in a solution of ^t^BuOH and deionized H_2_O (0.5 mL, v/v = 2/1) and stirred at rt for 10 min. Compound **4** (105 mg, 0.11 mmol, 2.5 equiv) and **mcp-D-click** (29 mg, 0.045 mmol, 1.0 equiv) dissolved in ^t^BuOH and deionized H_2_O (0.5 mL, v/v = 2/1) were added, and the reaction mixture was stirred at rt overnight. Excess of copper was removed by CuS-precipitation with Na_2_S (10 mg) and filtration. The solvent was removed under reduced pressure, and the residue was purified by reverse-phase column chromatography (H_2_O/acetonitrile +0.1% TFA; 90:10 → 55:45) to obtain compound **6** (29 mg, 27%) as a yellow oil after lyophilization. HRMS (ESI+) *m*/*z*: [M + Cu]^2+^ calcd for 1290.0512; found, 1290.0507.

### Synthesis of mcp-M-Sn-alb-PSMA (10)

Compound **5** (1.07 mg, 0.7 μmol, 1.0 equiv) was dissolved in DMSO (100 μL), and Et_3_N (15 μL) was added. After 10 min, compound **3** (0.28 mg, 0.63 μmol, 0.9 equiv) was added and the solution was stirred at 40 °C for 1 h. After complete conversion of compound **3** monitored by analytical HPLC, the solution was lyophilized and again dissolved in DMSO (final concentration 1 mM) and stored at −20 °C. MS (MALDI): *m*/*z* = 1835 [M + H]^+^; HRMS (ESI+): *m*/*z* calcd 1671.7941 [M-SnMe_3_+H]^+^; found, 1671.7943.

### Synthesis of mcp-D-Sn-alb-PSMA (12)

Compound **6** (1.2 mg, 0.5 μmol, 1.0 equiv) was dissolved in DMSO (100 μL), and Et_3_N (15 μL) was added. After 10 min, compound **3** (0.41 mg, 0.9 μmol, 1.9 equiv) was added, and the solution was stirred at 40 °C for 1 h. After complete conversion of compound **3** monitored by analytical HPLC, the solution was lyophilized and again dissolved in DMSO (final concentration 1 mM) and stored at −20 °C. MS (MALDI): *m*/*z* = 3137 [M + H]^+^; HRMS (ESI+): *m*/*z* calcd 1406.1691 [M-2SnMe_3_+2H]^2+^; found, 1406.1654.

### Radiolabeling with ^123^I

The production of [^123^I]­I^–^ was achieved by means of proton irradiation (30 MeV) of a KIPROS 200 xenon target (^124^Xe) at a TR-FLEX (ACSI) cyclotron at the Helmholtz–Zentrum–Dresden–Rossendorf. Further processing was carried out by ROTOP Pharmaka GmbH and was kindly supplied to us as a [^123^I]­NaI solution in 0.02 M NaOH.

A pierce iodination tube precoated with iodogen was rinsed with H_2_O (1 mL). In the reaction tube, phosphate buffer (0.18 M, pH 6, 350 μL), ethanol (90 μL), and the precursor (1 mM in DMSO, 10 μL) were added. The reaction was started by adding [^123^I]­NaI (180 μL, 5 GBq) and carried out for 25 min at room temperature. For **mcp-D-Sn-alb-PSMA**, a nonradioactive NaI solution (50 μL, 100 mM) was added after 20 min, and the reaction was further proceeded for 5 min. The solution was transferred to a glass vial (for nonradioactive La-complexation, an excess of La­(NO_3_)_3_ (50 μL, 100 mM) was added at this stage) and diluted with H_2_O/acetonitrile (0.6 mL, 3/1; v,v). Purification of the ^123^I-ligands was performed by semipreparative radio-HPLC: Jasco LC-NetII/ADC HPLC system with a GABI gamma spectrometer (Elysia-Raytest GmbH) with a Phenomenex Luna C18 column (10 μm, 250 × 10 mm) using a linear gradient from H_2_O/acetonitrile +0.1% TFA: 75/25 → 25/75 in 33 min, flow rate: 4 mL/min. The product fraction (855 MBq) was diluted with H_2_O (2 mL) and applied to a C18 cartridge. The product was eluted with ethanol (1 mL), and the solvent was evaporated under a N_2_ flow in vacuum. The residue (446 MBq) was taken up in 0.9% NaCl solution (5 mL) and analyzed via analytical radio-HPLC: Agilent 1200 HPLC system with a GABI gamma spectrometer (Elysia-Raytest GmbH). The column used was a Purospher RP-18 end-capped (5 μm, 125 × 3 mm). Elution was performed with a linear gradient from H_2_O/acetonitrile +0.1% TFA 90/10 → 5/95 in 15 min (total 30 min) at a flow rate of 1 mL/min.

### Radiolabeling with ^133^La

Lanthanum-133 was produced in-house according to previously published papers.^25,27^ For radiolabeling, the precursor dissolved in NH_4_OAc buffer (0.2 M, pH 6, final concentration 1 mM, 4 μL) was diluted with NH_4_OAc (0.2 M, pH 6, 50 μL). The reaction was started by adding [^133^La]­LaCl_3_ (150 μL, up to 200 MBq) and carried out for 30 min at room temperature for all macropa-derivatives and for 30 min at 90 °C for **PSMA-617.** Two radio-TLC systems were utilized in order to determine the radiochemical conversion (RCC). The first system involved the use of a solution of 7:3 acetonitrile/water as the solvent for silica gel 60 RP-18 F_254S_ TLC plates, while a 50 mM EDTA (pH 6.0) solution was employed as the solvent for silica gel 60 F_254_ TLC plates.

Analytical radio-HPLC was conducted using a Knauer/Azura 6.1 L HPLC system in conjunction with a GABI gamma spectrometer (Elysia-Raytest GmbH). The column employed was a Synergi Hydro-RP 80A LC column (4 μm, 250 × 4 mm). Elution was performed using a linear gradient from H_2_O/acetonitrile +0.05% acetic acid (100/0) to 30/70 over a period of 20 min at a constant flow rate of 1 mL/min.

For all experiments, the radioligand was used without further purification.

### Serum Stability

**mcp-M-alb-PSMA** (2 nmol) was radiolabeled with [^133^La]­La^3+^ (100 MBq) at rt for 30 min to obtain **[** ^**133**^ **La]­La-mcp-M-alb-PSMA** in a radiochemical conversion of >99%. The radioligand (50 μL) was added to 1 mL of human serum. The resulting solution was shaken at 37 °C. Subsequent to the designated time points (1, 4, and 24 h), the stability of the radioconjugate was evaluated by radio-HPLC. For this, 50 μL (1, 4, and 24 h: 100 μL) of the solution was treated with 100 μL of acetonitrile, leading to the precipitation of the human serum. After centrifugation, 10 μL of the supernatant was diluted in 100 μL of water and subjected to radio-HPLC analyses. No proteolytic degradation was observed after 24 h.

### Cell Culture

The PSMA-positive human prostate adenocarcinoma cell line LNCaP was obtained from ATCC (Manassas, VA, USA) and routinely cultured in RPMI-1640 medium (Thermo Fisher Scientific, Waltham, MA, USA) supplemented with 10% fetal calf serum (FCS, Merck KGaA, Darmstadt, Germany) as previously reported.^29,30^

### *In Vitro* Characterization

In order to determine the PSMA-binding affinity, a competitive cell binding assay was performed according to the general protocol provided by Bigott-Hennkens et al. with a few modifications.^39^ Briefly, LNCaP cells (80000/well/250 μL) were seeded in 48-well microplates and cultivated for 48 h to allow cell adhesion and growth. Cell culture media was removed and replaced with RPMI-1640 medium supplemented with 0.001% bovine serum albumin containing 16 different concentrations of **mcp-M-alb-PSMA**, **La-mcp-M-alb-PSMA**, **mcp-D-alb-PSMA,** or **La-mcp-D-alb-PSMA** (0–10000 nM, 100 μL/well). **[** ^**133**^ **La]­La-PSMA-617** (2 nM, 100 μL/well) was added to each well (in triplicate) resulting in a final radioligand concentration of 1 nM and in final nonradioactive ligand concentrations of 0–5000 nM. After incubation at 37 °C for 1 h with gentle agitation, media and radioligand were removed, and cells were washed three times with ice-cold Dulbecco’s PBS with 0.5 mM MgCl_2_ and 0.9 mM CaCl_2_ (400 μL/well). Finally, the cells were lysed by the addition of 1% SDS/0.1 M NaOH (200 μL/well) and incubated for 30 min at rt with vigorous shaking. To quantify the radioactivity in the cell lysates, an automatic gamma counter (Hidex Deutschland Vertrieb GmbH, Mainz, Germany) was used. The total protein concentration in cell extracts was determined using the DC Protein Assay (Bio-Rad Laboratories GmbH, Feldkirchen, Germany) according to the manufacturer’s microplate assay protocol using bovine serum albumin as protein standard. The equilibrium dissociation constant *K*_i_ for each competitor was determined from the measured data using a nonlinear regression algorithm of the Prism software (Version 10, GraphPad).

The extent of internalization was determined for each ^133^La-labeled compound based on the general protocol provided by Bigott-Hennkens et al. with a few modifications.^39^ In short, LNCaP cells (60000/well/250 μL) were seeded in 48-well microplates and cultured overnight. After replacement of the cell culture media with RPMI-1640 medium supplemented with 0.001% bovine serum albumin (100 μL/well), the cells were incubated with the ^133^La-labeled compounds (10 nM final concentration) for up to 4 h at 37 °C (in triplicate). To determine PSMA-specific uptake, cells were blocked with unlabeled **PSMA-617** to a final concentration of 500 μM. Internalization was terminated by washing with ice-cold Dulbecco’s PBS with 0.5 mM MgCl_2_ and 0.9 mM CaCl_2_ (400 μL/well) for 5 min on ice. To remove surface-bound radioligands, cells were incubated twice with ice-cold glycine-HCl (50 mM, pH 2.8, 400 μL/well) for 5 min on ice. Afterward, the cells were washed with ice-cold Dulbecco’s PBS with 0.5 mM MgCl_2_ and 0.9 mM CaCl_2_ (400 μL/well) for 5 min on ice and lysed with 1% SDS/0.1 M NaOH (200 μL/well). The radioactivity of the surface-bound and internalized fractions was measured using an automatic gamma counter (Hidex Deutschland Vertrieb GmbH, Mainz, Germany), and their total protein concentration was determined using the DC Protein Assay (Bio-Rad Laboratories GmbH, Feldkirchen, Germany) according to the manufacturer’s microplate assay protocol using bovine serum albumin as protein standard.

### Animal Experiments

All animal experiments were carried out according to the guidelines of the German Regulations for Animal Welfare and have been approved by the local Ethical Committee for Animal Experiments (reference number: DD24.1–5131/499/49). A prostate cancer xenograft model was generated via subcutaneous injection of 5 × 10^6^ human LNCaP cells into the right shoulder of 8–12 week old male nude mice (Rj:NMRI-*Foxn1* ^nu/nu^, Janvier Laboratories, Le Genest-Saint-Isle, France). Imaging studies were performed when tumors had reached a diameter of >6 mm. For imaging studies, anesthesia was induced and maintained with inhalation of 10% (v/v) desflurane in 30/10% (v/v) oxygen/air. During anesthesia, animals were continuously warmed at 37 °C. Small blood samples were collected from anesthetized animals via retrobulbar sampling. Final blood samples were collected from anesthetized animals via cardiac puncture, followed by immediate sacrifice using CO_2_ inhalation and cervical dislocation.

### Small-Animal PET Imaging

Small-animal PET imaging was performed using the nanoScan PET/CT scanner (Mediso Medical Imaging Systems, Budapest, Hungary). A 5 mL syringe filled with 2 mL of Dulbecco’s phosphate-buffered saline containing 15 MBq of [^133^La]­La^3+^ served as resolution and activity phantom. Each animal received 15–25 MBq (eqv. to 0.1–0.3 nmol) of the lanthanum-133-labeled PSMA radioligands delivered in Dulbecco’s phosphate-buffered saline via intravenous injection through a lateral tail vein catheter within the initial 30 s after scan start. Emission of photons was continuously recorded during the time windows 0–2, 3.5–4.5, and 21–24 h after radioligand injection at a coincidence mode of 1:5. With each scan, a corresponding CT image was captured and used for anatomical referencing and attenuation correction. Binning and time framing were performed as reported previously.^40^ Images were reconstructed using the Tera-Tomo three-dimensional (3D) algorithm with a voxel size of 0.4 mm, applying corrections for attenuation, scattering, and decay.

### Small-Animal SPECT Imaging

Quantitative SPECT imaging was performed using the nanoScan SPECT/CT scanner (Mediso Medical Imaging Systems). A 5 mL syringe filled with 2 mL of Dulbecco’s phosphate-buffered saline containing 15 MBq of [^123^I]­I^–^ served as resolution and activity phantom. Each animal received 20–30 MBq (eqv. to <0.03 nmol) of the iodine-123-labeled PSMA radioligands delivered in Dulbecco’s phosphate-buffered saline via intravenous injection through a lateral tail vein catheter. SPECT images were acquired using the APT56 aperture consisting of four M3 multipinhole ultrahigh-energy (UHE) collimators. Emission of photons was continuously recorded during the time windows 0.75–1.25, 2.75–3.25, 18.5–19.5, and 43–45 h after radioligand injection and binned within the 20% energy window of the 159 keV photopeak. With each scan, a corresponding CT image was captured and used for anatomical referencing and attenuation correction. Images were reconstructed using the Tera-Tomo 3D algorithm with a voxel size of 0.23 mm, applying corrections for attenuation, scattering, collimator plate scattering, and decay.

### Image Processing and Analysis

PET and SPECT images were postprocessed and analyzed using Rover version 3.0.77 h (ABX GmbH, Radeberg, Germany) and displayed as maximum intensity projections with common scale over indicated time points. From phantom scans, the spatial resolution of PET and SPECT images was determined using the “Resolution tool” implemented in Rover as described elsewhere.^41,42^ The activity values of voxel intensities were calibrated by determining the conversion factor between the activity (MBq) of the syringe content measured in an ISOMED 2010 dose calibrator (NUVIA Instruments, Dresden, Germany) and the corresponding counts per second (cps) in a reconstructed image with a 50 mm axial field-of-view.

For extraction of region-averaged standardized uptake values (SUV_mean_), from animal images, regions of interest (ROIs) were generated within spherical preselection masks including voxels with intensities above indicated tissue-specific thresholds (% of maximum voxel intensity) as follows: heart (>60%, 30 mm^3^ of blood content), kidneys (>50%, 220 mm^3^ of cortical regions), lung (>0%, 10 mm^3^ of the left lobe), liver (>0%, 30 mm^3^ of a central lobe), muscle (0%, 30 mm^3^ of triceps brachii), tumor (>39%), parotid glands (>50%, 30 mm), urinary bladder (>10%), and total body (>1%, urinary bladder excluded). Examples of ROIs are provided in the Supporting Information (Figure S4). Time courses of uptake values in blood and total body were analyzed by nonlinear regression using the “two-phase decay” and “one-phase decay” models implemented in Prism 10 (GraphPad Software, San Diego, CA, USA). Total activity amounts in the liver were extrapolated from the regional activity concentration using a realistic dosimetry model for 35 g mice.^43^ Theoretical initial uptake values in mouse blood (SUV_mean,blood_ (*t*_0_)) were calculated from animal body weight (BW), administered volume of radiotracer (*V*_RT_ = 0.2 mL), and published data for total blood volume per body weight of animals (*V*_Blood_ = 0.074 mL/g [51]), assuming a tissue density of 1 g/mL (1 g = 1 mL). SUV_mean,blood_ (*t*_0_) = (BW [g] + *V*_RT_ [mL])/(0.074 [mL/g] × BW [g] + *V*_RT_ [mL]).

### Metabolite Analysis

An ex vivo metabolite analysis was performed after intravenous injection of **La-mcp-M-[** ^**123**^ **I]­alb-PSMA** and **[** ^**133**^ **La]­La-mcp-M-alb-PSMA** in healthy mice. Blood samples were collected and centrifuged at 16,100 g for 2 min to obtain plasma. The supernatant was diluted (1:1, v/v) with Supersol (20% ethanol, 0.5% Triton X-100, 5 mM EDTA, 0.5 mM o-phenanthroline, and 0.1% saponin)^44^ and centrifuged again. The resulting supernatant was analyzed by radio-HPLC.

Urine samples were collected directly from the urinary bladder after euthanasia. The urine was diluted (1:1, v/v) with Supersol and centrifuged. The supernatant was analyzed by a radio-HPLC.

Kidneys were excised and homogenized in Supersol, and after centrifugation, the supernatant was analyzed by radio-HPLC. No significant activity remained in the precipitate.

## Supplementary Material





## Notes

Saturation binding of [^133^La]­La-PSMA-617; cell studies for saturation assay; decay-corrected activity concentrations of ^133^La- and ^123^I-labeled PSMA ligands; influence of the radiotracer preparation on the blood kinetics of ^133^La- and ^123^I-labeled PSMA radiohybrid ligands; extraction of tissue-specific uptake values from PET and SPECT images; radiometabolite analysis; syntheses of the stannyl intermediates; compound characterization data; and radio-HPLC analyses (PDF)
Molecular formula strings (CSV)

## Abbreviations

DOTA: 1,4,7,10-tetraazacyclododecane-1,4,7,10-tetraacetic acid
EDTA: ethylenediaminetetraacetic acid
LNCaP: Lymph Node Carcinoma of the Prostate
mCRPC: metastatic castration-resistant prostate cancer
PET: positron emission tomography
PSMA: prostate-specific membrane antigen
SPECT: single-photon emission computer tomography
SUV: standard uptake value
TAT: targeted alpha therapy
THPTA: tris­(3-hydroxypropyltriazolylmethyl)­amine
