# Supplementary material for: Diagnostic Twins: Exploring the Radiohybrid Concept with Iodine-123 and Lanthanum-133 for PSMA-Targeted SPECT and PET Imaging
Source: J Med Chem. 2026 Apr 16;69(9):11072–87. doi: 10.1021/acs.jmedchem.6c00161 (PMC13181793; doi:10.1021/acs.jmedchem.6c00161)

Diagnostic Twins: Exploring the Radiohybrid Concept with Iodine−123 and Lanthanum−133 for PSMA-Targeted SPECT and PET Imaging

Tobias Krönke[a,#], Martin Ullrich[a,#,*], Magdalena K. Blei[a,b], Kristof Zarschler[a], Jonas Schädlich[a,b], Santiago Andrés Brühlmann[a], Klaus Kopka[a,b,c,d], Jens Pietzsch[a,b], Sven Stadlbauer[a,b], Constantin Mamat[a,b,*]

a Helmholtz-Zentrum Dresden-Rossendorf, Institute of Radiopharmaceutical Cancer Research, Bautzner Landstraße 400, D-01328 Dresden, Germany.

b TU Dresden, School of Science, Faculty of Chemistry and Food Chemistry, D-01062 Dresden, Germany.

c National Center for Tumor Diseases (NCT), NCT/UCC Dresden, a partnership between DKFZ, Faculty of Medicine and University Hospital Carl Gustav Carus, TU Dresden & Helmholtz-Zentrum Dresden-Rossendorf (HZDR), D-01307 Dresden, Germany.

d German Cancer Consortium (DKTK), Partner Site Dresden, and German Cancer Research Center (DKFZ), D-69120 Heidelberg, Germany.

[#] These authors contributed equally to this work.

Corresponding authors: Dr. Constantin Mamat (c.mamat@hzdr.de); Dr. Martin Ullrich (m.ullrich@hzdr.de)

KEYWORDS. Theranostic concept, radiohybrid approach, targeted alpha therapy, lanthanum-133

Table of Contents

Saturation Binding of **[^{133}La]La-PSMA-617** …………………………………………………….. 3

Cell studies for saturation assay ……………………………………………………………….. 4

Decay-corrected activity concentrations of ^{133}La- and ^{123}I-labeled PSMA ligands ……………………. 5

Influence of the radiotracer preparation on the blood kinetics of ^{133}La- and ^{123}I-labeled PSMA radiohybrid ligands …………………………………………………………………….…..... 6

Extraction of tissue-specific uptake values from PET and SPECT images ……………………………. 7

Radiometabolite analysis ………………………………………………………………………. 8

Syntheses of the stannyl intermediates ………………………………………………………… 9

Compound characterization data ……………………………………………………………... 10

Mass spectra of compounds ……………………………………………………………………... 14

Radio-HPLC analyses ………………………………………………………………………...…. 19

Saturation Binding of [^{133}La]La-PSMA-617

Cell binding studies of **[^{133}La]La-PSMA-617** were performed using the PSMA-expressing cell line LNCaP. Binding of the radioligand to the cells was blocked in the presence of an excess of unlabeled **PSMA-617** confirming its target specificity. The equilibrium dissociation constants (K_d) was calculated by the GraphPad Prism software using a "One site–total and nonspecific binding" analysis (Figure S1).

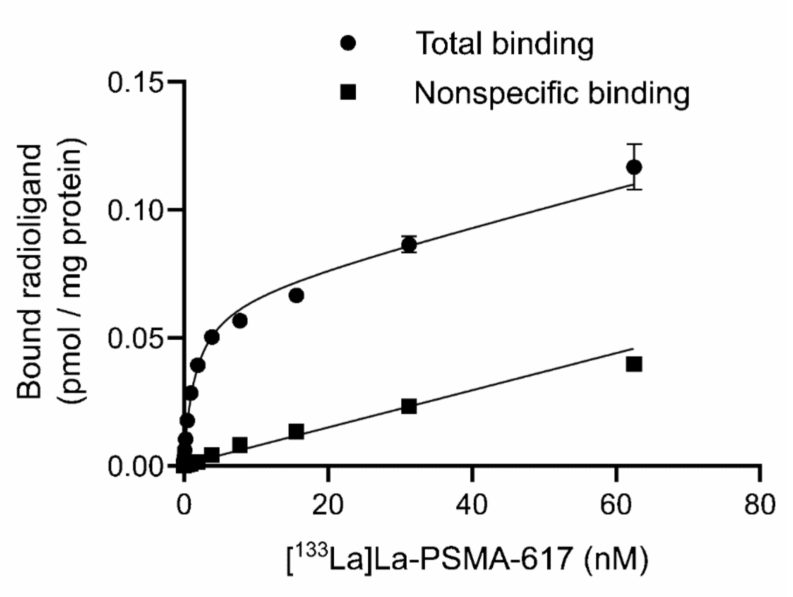

Figure S1. Saturation binding curves for **[^{133}La]La-PSMA-617** using PSMA-positive LNCaP cells. Nonspecific binding was determined in the presence of 500 μM of unlabeled **PSMA-617**.

A K_d of 1.5 nM was determined for **[^{133}La]La-PSMA-617**, which corresponds with those of **[$^{nat/68}$Ga]Ga-PSMA-617**, **[$^{nat/44}$Sc]Sc-PSMA-617** and **[$^{nat/177}$Lu]Lu-PSMA-617**, respectively.

Benešová M, et al. Preclinical Evaluation of a Tailor-Made DOTA-Conjugated PSMA Inhibitor with Optimized Linker Moiety for Imaging and Endoradiotherapy of Prostate Cancer. J Nucl Med. 2015; 56: 914–920.

Eppard E, et al. Clinical Translation and First In-Human Use of [^{44}Sc]Sc-PSMA-617 for PET Imaging of Metastasized Castrate-Resistant Prostate Cancer. Theranostics. 2017; 7: 4359–4369.

Eder M, et al. ^{68}Ga-complex lipophilicity and the targeting property of a urea-based PSMA inhibitor for PET imaging. Bioconjug Chem. 2012; 23: 688-697.

Cell studies for saturation assay

Cell binding studies were performed as previously reported with slight modifications [37]. LNCaP cells were plated in 48-well microplates at a density of 50000 cells/200 µL/well and incubated for 48 h. During the binding experiment, cells were kept on ice and all reagents were added ice-cold. The cell culture medium was replaced by Dulbecco's PBS with 0.5 mM $MgCl_2$ and 0.9 mM $CaCl_2$ (500 µL/well) and the microplates were preincubated at 4 °C. After 10 min, the buffer was replaced by 200 µL Dulbecco's PBS with 0.5 mM $MgCl_2$ and 0.9 mM $CaCl_2$ (total binding) or 200 µL Dulbecco's PBS with 0.5 mM $MgCl_2$, 0.9 mM $CaCl_2$ and 1 mM unlabeled **PSMA-617** (nonspecific binding), respectively. The radiolabeled ligand was step-wise diluted to 16 different concentrations ranging from 0.03 nM to 1 µM with Dulbecco's PBS with 0.5 mM $MgCl_2$, 0.9 mM $CaCl_2$ and 2 % bovine serum albumin. Then, 200 µL of these dilutions were added to the wells resulting in final radioligand concentrations of 0.015 nM to 500 nM, and the cell culture microplates were further incubated for 90 min on ice. After incubation, the cells were washed three times with ice-cold Dulbecco's PBS with 0.5 mM $MgCl_2$ and 0.9 mM $CaCl_2$ (500 µL/well). Finally, the cells were lysed by the addition of 1 % SDS/0.1 M NaOH (250 µL/well) and incubation for 30 min at room temperature with vigorous shaking. To quantify the radioactivity in the cell lysates, an automatic gamma counter (Hidex Deutschland Vertrieb GmbH, Mainz, Germany) was used. The total protein concentration in cell extracts was determined using the DC Protein Assay (Bio-Rad Laboratories GmbH, Feldkirchen, Germany) according to the manufacture's microplate assay protocol using bovine serum albumin as protein standard. The equilibrium dissociation constant K_d was determined from the measured data using the Prism software (version 10, GraphPad).

Decay-corrected activity concentrations of ^{133}La- and ^{123}I-labeled PSMA ligands

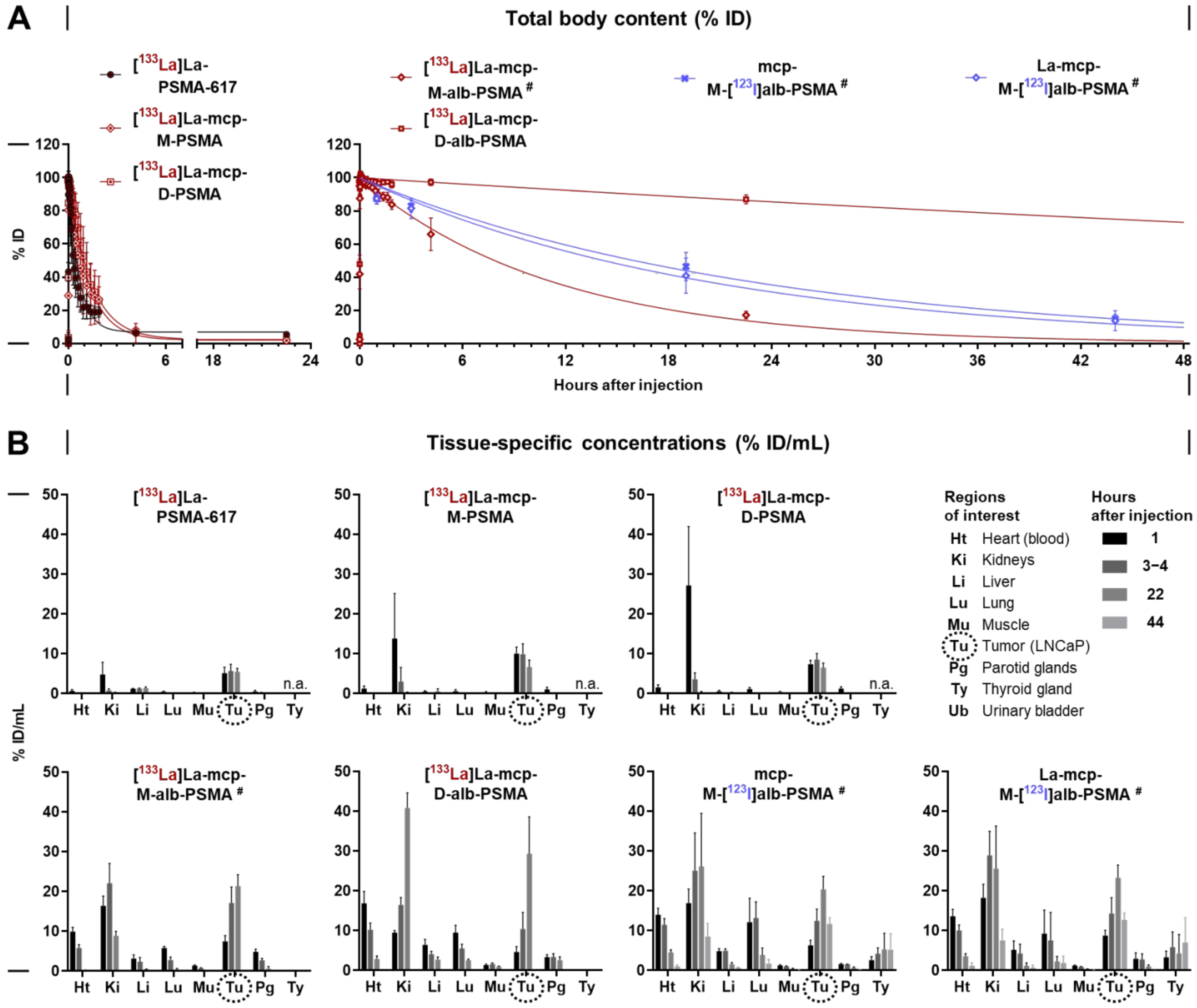

Figure S2. Distribution of ^{133}La- and ^{123}I-labeled PSMA radioligands in LNCaP tumor-bearing mice determined by quantitiatve analysis of PET and SPECT images; (A) Time-resolved changes in the total activity content of the body, excluding tumor and urinary bladder; data fitted with the 'one-phase decay' non-linear regression model; total body half-lives are provided in **Error! Reference source not found.**; (B) Region-averaged activity concentrations in specific tissues at indicated time points after radioligand injection; data presented as decay-corrected mean values with standard deviation; numbers of replicates and experiments are provided in **Error! Reference source not found.**; (% ID) percent of initially injected activity dose; # indicates corresponding radiohybrid ligands.

Influence of the radiotracer preparation on the blood kinetics of ^{133}La- and ^{123}I-labeled PSMA radiohybrid ligands

As tested for the monovalent, bispecific PET radiotracer **[^{133}La]La-mcp-M-alb-PSMA**, its preparation using the same radiolabeling matrix and procedure as for the corresponding SPECT radiotracer **La-mcp-M-[^{123}I]alb-PSMA** (SPE extraction, elution with EtOH) did not change the kinetic profile in blood (Figure S3A). This indicates that, e.g., interactions of the vacant coordination site in the lanthanum-macropa complex with the buffer ions, drying, and reconstitution of the radiolabeled product in ethanol have no influence on the blood half-lives of the radiohybrid ligands.

As tested for the monovalent, bispecific SPECT radiotracer **La-mcp-M-[^{123}I]alb-PSMA**, two preparations with different molar activities (n.c.a. versus 50 MBq/nmol) showed the same kinetic profile in blood (**Error! Reference source not found.**B, Figure S3B). This suggests that these differences in the molar activities and thus the administered substance amounts of **La-mcp-M-[^{123}I]alb-PSMA** (typically n.c.a, < 0.03 nmol/animal) compared to chemically identical PET radiotracer **[^{133}La]La-mcp-M-alb-PSMA** (typically 30−50 MBq/nmol, 0.1−0.3 nmol/animal) may also have no influence on the blood half-lives of the radiohybrid ligands.

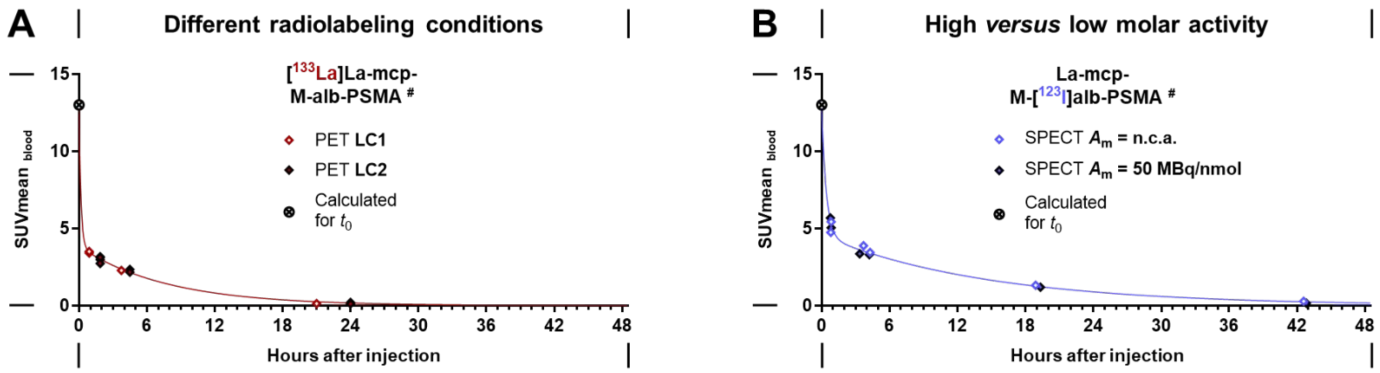

Figure S3. Excluding the influence of the radiotracer preparation on blood kinetics of ^{133}La- and ^{123}I-labeled PSMA radiohybrid ligands; time-resolved changes in image-derived uptake values extracted from the blood content of the heart; (A) Uptake values of **[^{133}La]La-mcp-M-PSMA** in blood remain unchanged, regardless of different matrices and procedures used for radiolabeling; labeling conditions: LC1; LC2; (B) Uptake values of **La-mcp-[^{123}I]alb-M-PSMA** in blood remain unchanged, regardless of the molar activity of the radiotracer preparation; (t_0) time point of radioligand injection, theoretical initial uptake values in mouse blood were calculated based on published data for total blood volume per body weight (see methods section for details).

Extraction of tissue-specific uptake values from PET and SPECT images

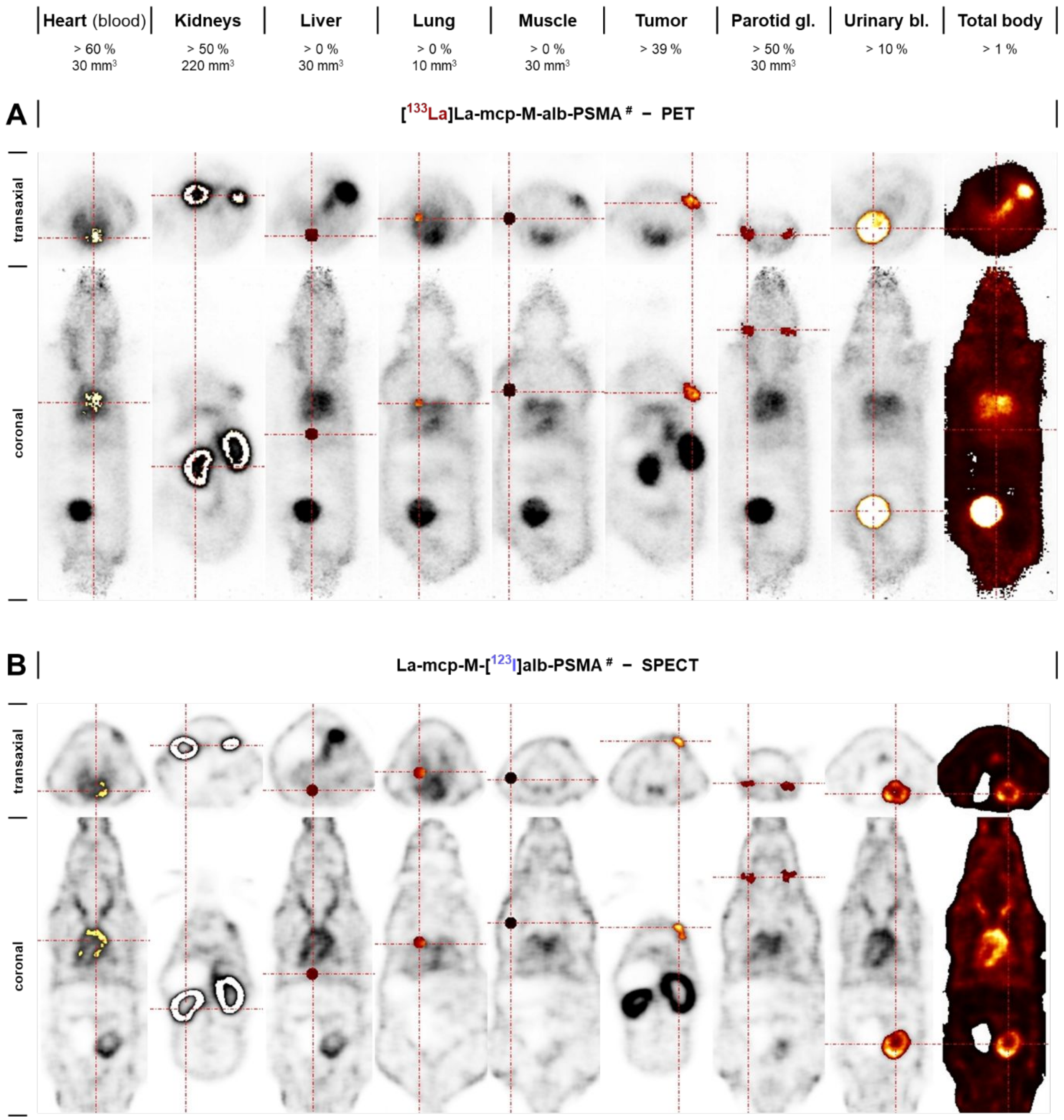

Figure S4. Regions-of-interest drawn in Rover for extraction of tissue-specific uptake values form images of radioligand distribution in LNCaP-tumor-bearing mice; (A) Tissue delineation in PET images of [^{133}La]La-mcp-M-alb-PSMA, integrated signals from 0−2 h; (B) Tissue delineation in SPECT images of La-mcp-M-[^{123}I]alb-PSMA, integrated signals from 0.75−1.25 h; three-dimensional regions-of-interest (colored regions) drawn by including voxels with intensities above indicated tissue-specific thresholds (% of maximum voxel intensity), images presented as orthogonal slices centered at the indicated tissues (red crosshairs); # indicates corresponding radiohybrid ligands.

Radiometabolite Analysis

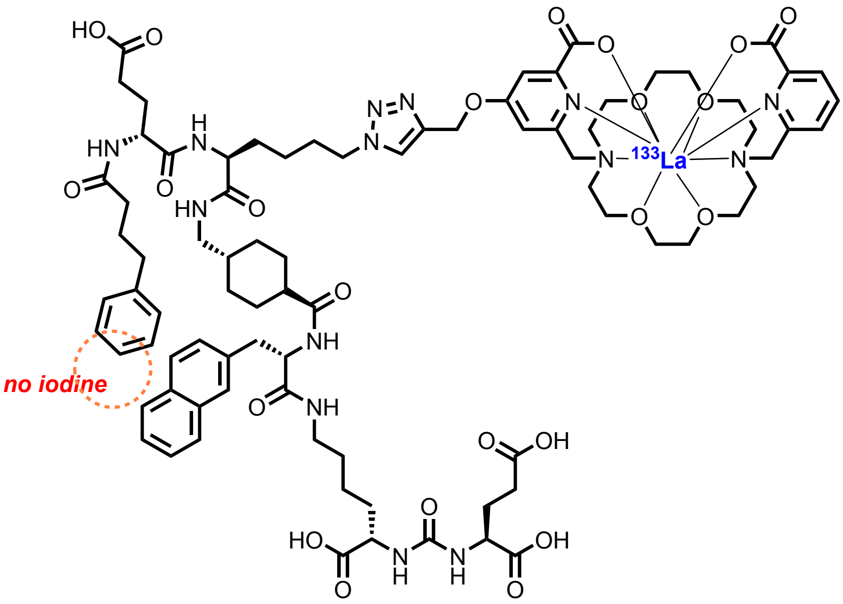

Figure S5. Chemical structure of the identified radiometabolite without iodine at the albumin binder.

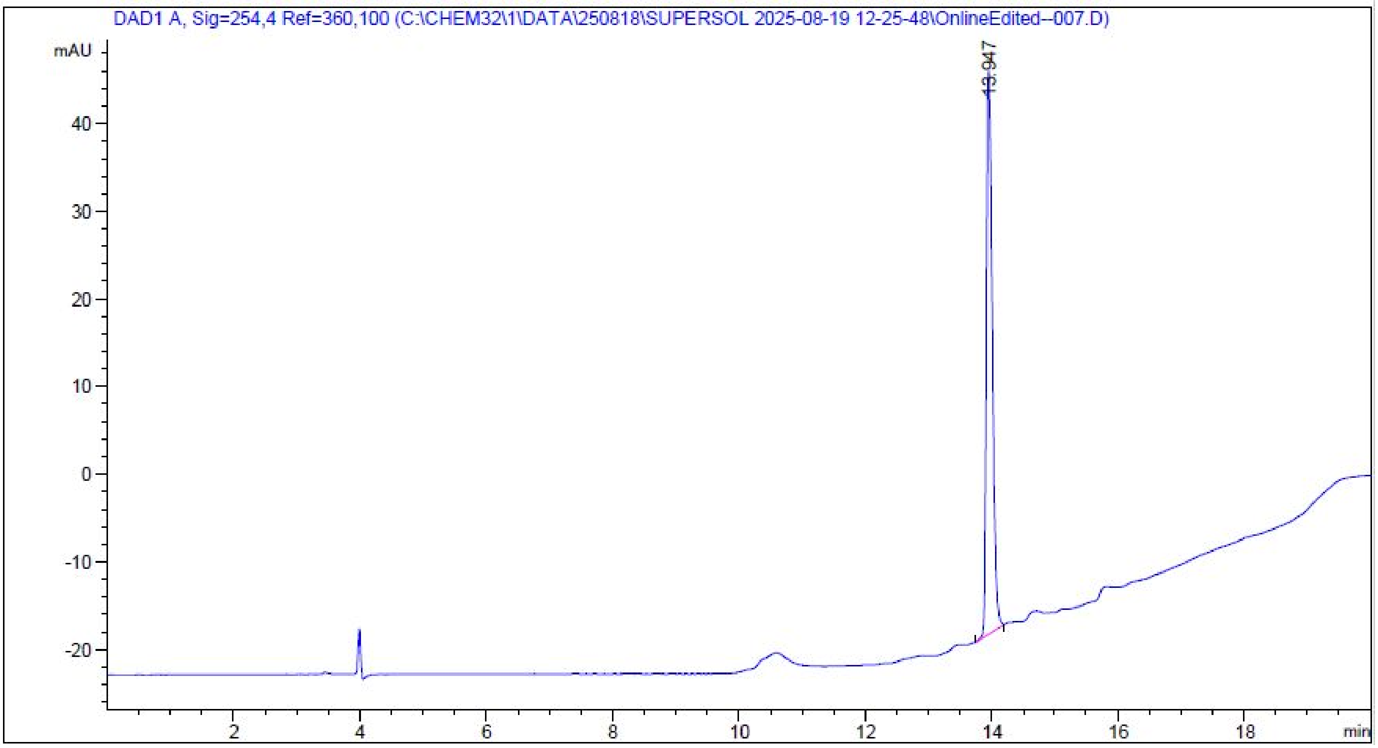

Figure S6. HPLC-chromatogram showing the radiometabolite of Figure S5 at t_R = 13.8 min.

Syntheses of the stannyl intermediates

Synthesis of 4-(4-(trimethylstannyl)phenyl)butyric acid (6).

4-(*p*-Iodophenyl)butyric acid (319 mg, 1.1 mmol, 1 eq.) was dissolved in anhydrous toluene (10 mL) under an argon atmosphere. Hexamethylditin (720 mg, 2.2 mmol, 2 eq.) and tetrakis(triphenyl-phosphane)palladium(0) (127 mg, 0,11 mmol, 0.1 eq.) were added and the resulting solution was stirred at 100 °C for 4 h. The solvent was removed and the crude product was purified by automated preparative column chromatography (silica, eluent: petroleum ether/ethyl acetate 100/0 → 85/15) to obtain **2** (160 mg, 40 %) as a colorless oil. ^{1}H NMR (400 MHz, $CDCl_3$): δ = 7.41 (d, ^{3}J = 7.8 Hz, 2H, H_{Ar}), 7.16 (d, ^{3}J = 7.8 Hz, 2H, H_{Ar}), 2.64 (t, ^{3}J = 7.6 Hz, 2H), 2.39 (t, ^{3}J = 7.5 Hz, 2H), 1.98 (q, ^{3}J = 7.6 Hz, 2H), 0.27 (t, $^{2}J_{H,Sn}$ = 27 Hz, 9H, CH_3) ppm; ^{13}C NMR (101 MHz, $CDCl_3$): δ = 180.4, 141.5, 128.6, 126.1, 35.3, 33.7, 26.8, 4.4, -2.3 ppm; ^{119}Sn NMR (224 MHz, $CDCl_3$): δ = -27.3 ppm.

Synthesis of 4-nitrophenyl 4-(4-(trimethylstannyl)phenyl)butanoate (7).

Compound **2** (280 mg, 0.85 mmol, 1 eq.) was dissolved in anhydrous dichloromethane (20 mL) and *p*-nitrophenol (143 mg, 1.0 mmol, 1.2 eq.) and *N,N'*-dicyclohexylcarbodiimide (265 mg, 1.2 mmol, 1.4 eq.) were added and the mixture was stirred at rt overnight. The solution was filtered and the solvent removed. The crude product was purified by automated preparative column chromatography (silica, eluent: petroleum ether/ethyl acetate 95/5 → 70/30) to obtain compound **3** (140 mg, 36 %) as colorless solid. ^{1}H NMR (400 MHz, $CDCl_3$): δ = 8.26 (d, ^{3}J = 9.1 Hz, 2H, H_{Ar}), 7.45 (d, ^{3}J = 7.8 Hz, 2H, H_{Ar}), 7.24 (d, ^{3}J = 9.0 Hz, 2H, H_{Ar}), 7.20 (d, ^{3}J = 7.8 Hz, 2H, H_{Ar}), 2.74 (t, ^{3}J = 7.5 Hz, 2H), 2.63 (t, ^{3}J = 7.5 Hz, 2H), 2.10 (p, ^{3}J = 7.7 Hz, 2H), 0.29 (s, 9H, CH_3) ppm; ^{13}C NMR (400 MHz, $CDCl_3$): δ = 171.1 (C=O), 155.5, 136.2, 128.5, 125.3, 122.6, 35.1, 33.8, 26.3, -9.4 ppm; ^{119}Sn NMR (400 MHz, $CDCl_3$): δ = -27.1 ppm.

Synthesis of compound 8

The ^{t}Bu-protected compound **^{t}Bu-4** was prepared as previously published.[2] For deprotection, **^{t}Bu-4** (155 mg, 0.13 mmol, 1.0 eq.) was dissolved in $CHCl_3$, TFA (3.0 mL, 12 mmol, 90 eq.) was added and the mixture was stirred at rt overnight. The solvent was removed and the crude product was purified by semi-preparative HPLC (H_2O/acetonitrile + 0.1 % TFA; 95:5 → 5:95) to obtain **4** (83 mg, 68 %) as yellow solid after lyophilization. HRMS (ESI+): *m/z* = calc. 939.4570 $[M+H]^+$, found 939.4552.

Compound characterization data

4-(4-(Trimethylstannyl)phenyl)butyric acid (6)

^{1}H NMR spectrum

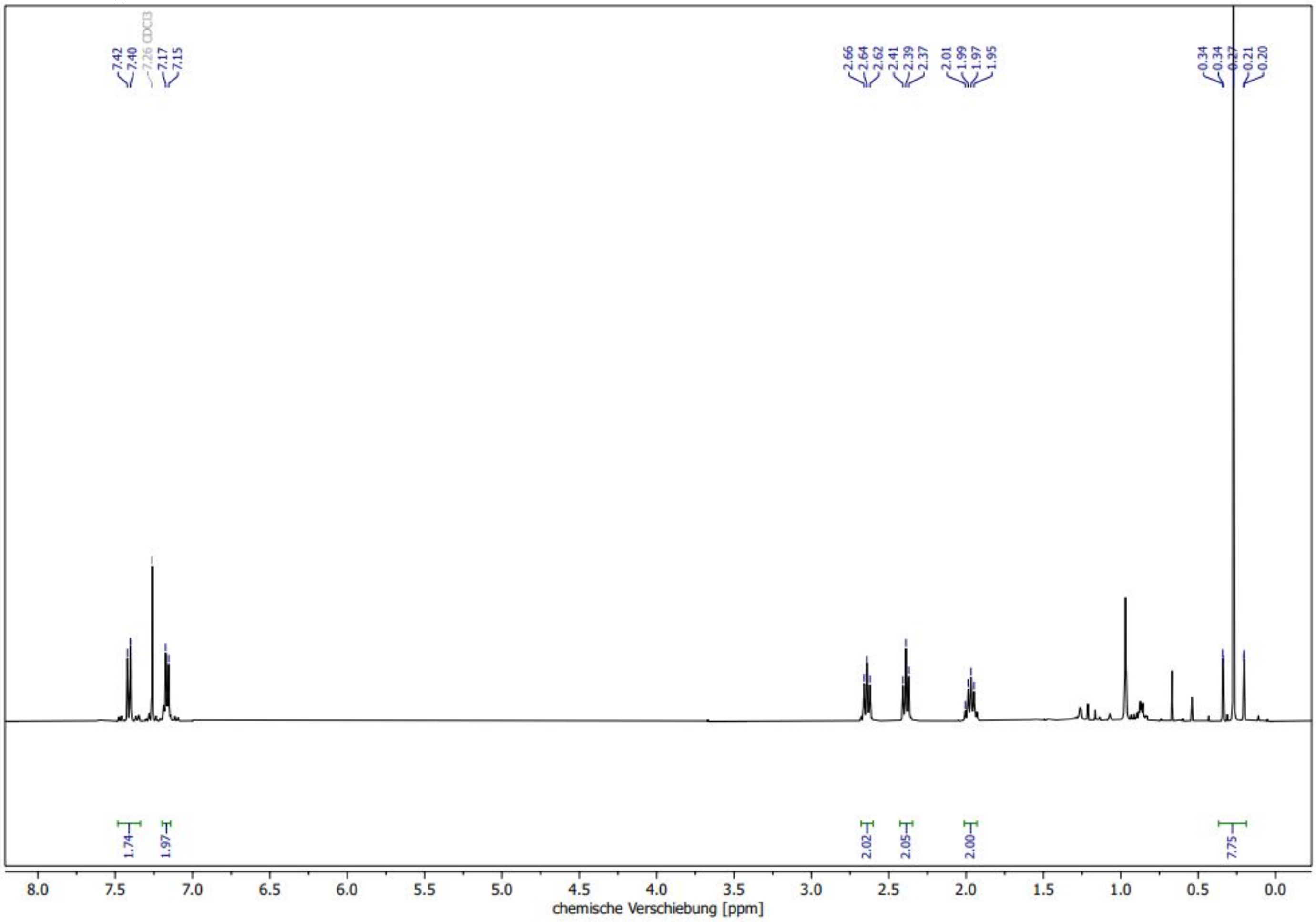

^{13}C NMR spectrum

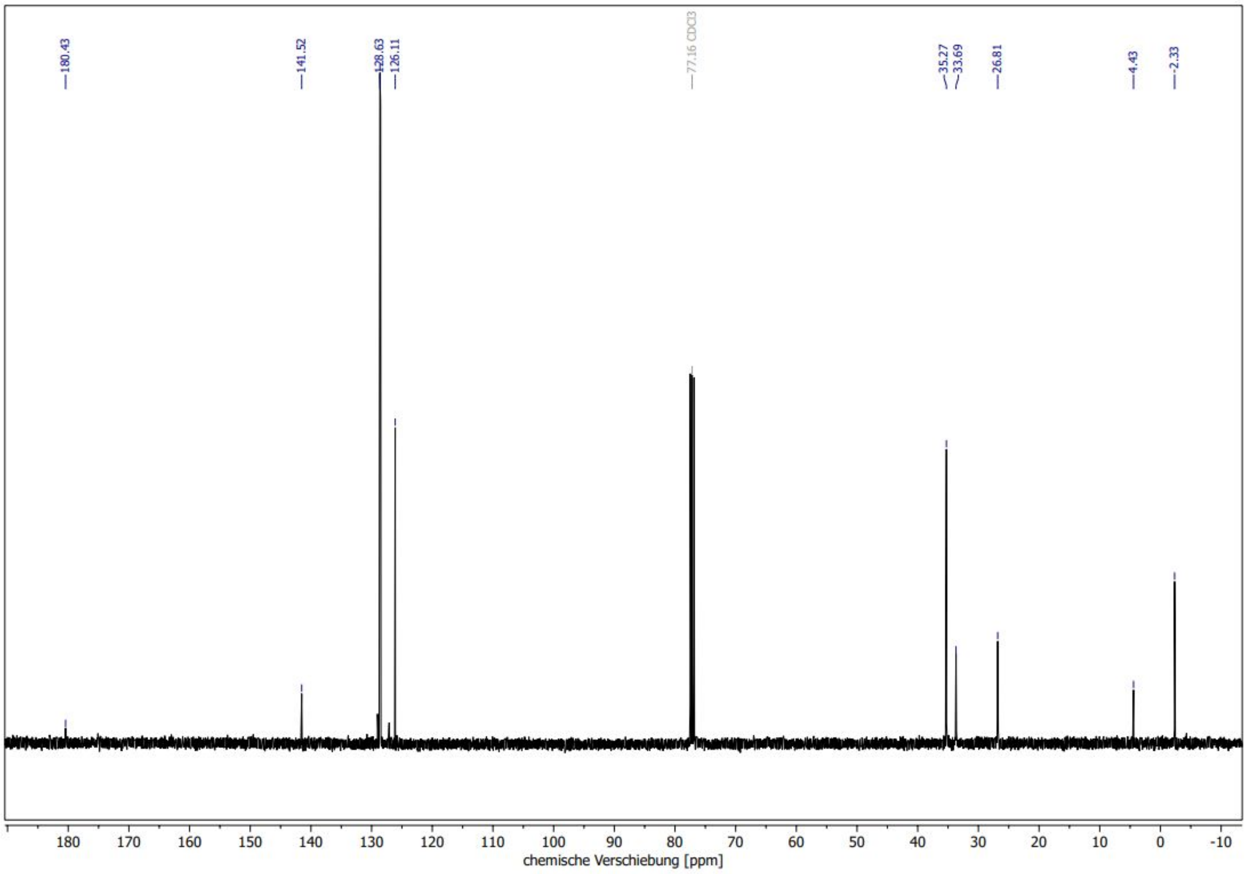

^{119}Sn NMR spectrum

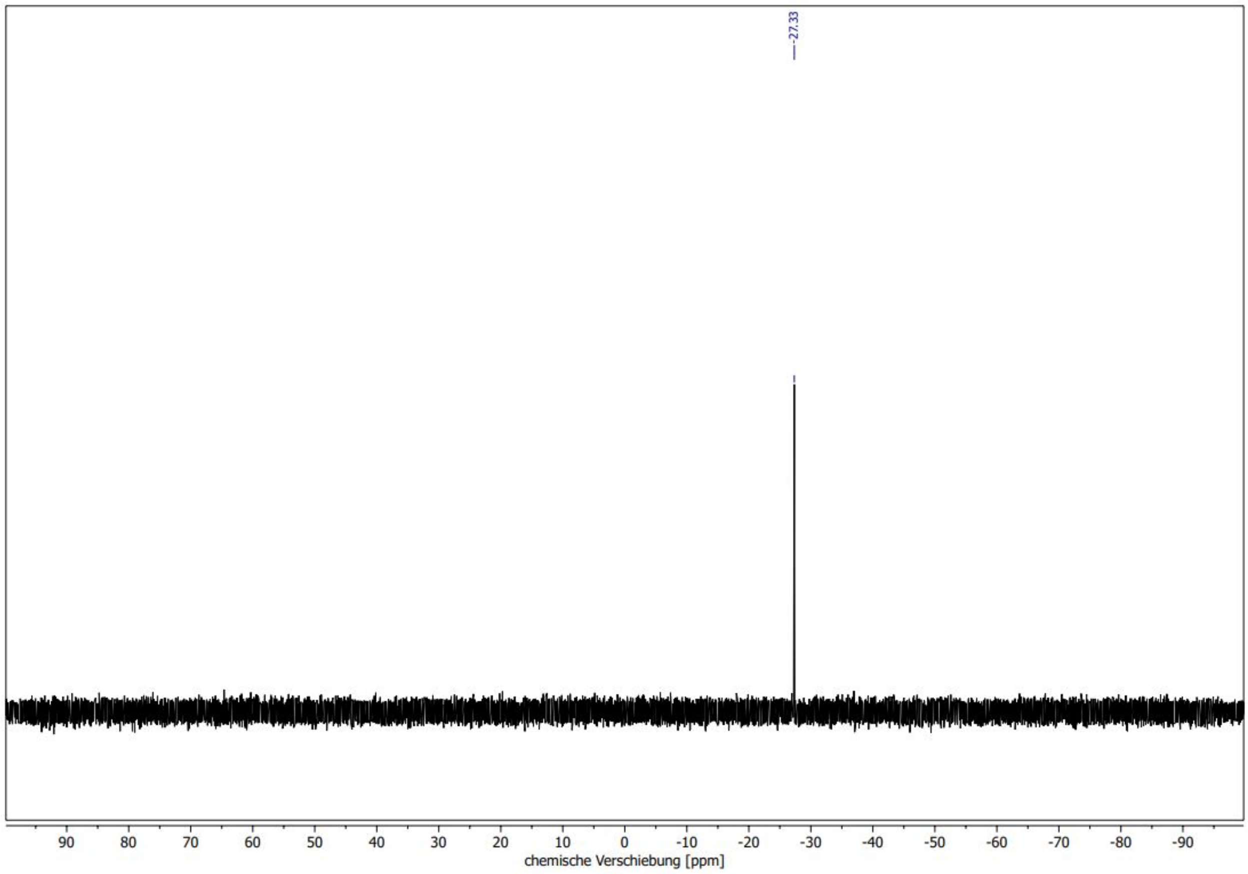

4-Nitrophenyl 4-(4-(trimethylstannyl)phenyl)butanoate (7)

^{1}H NMR spectrum

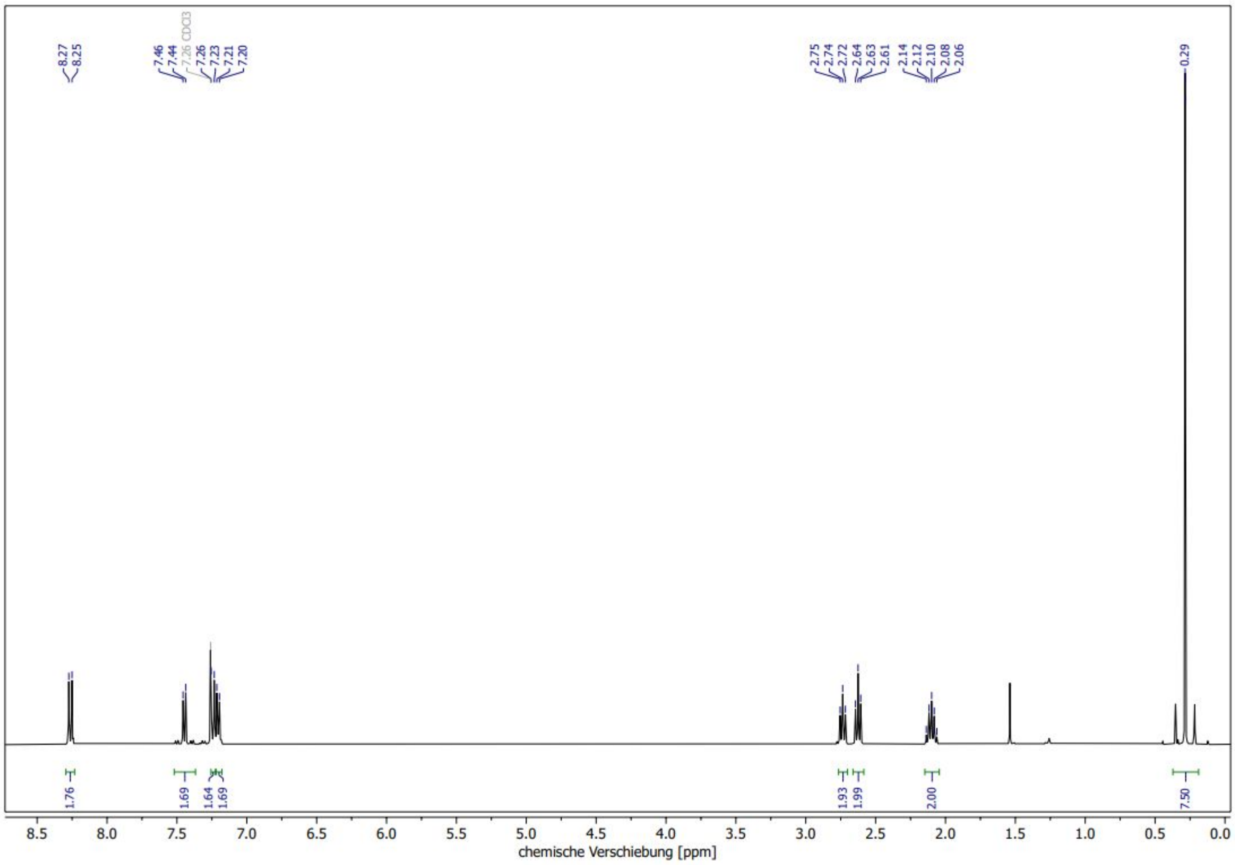

^{13}C NMR spectrum

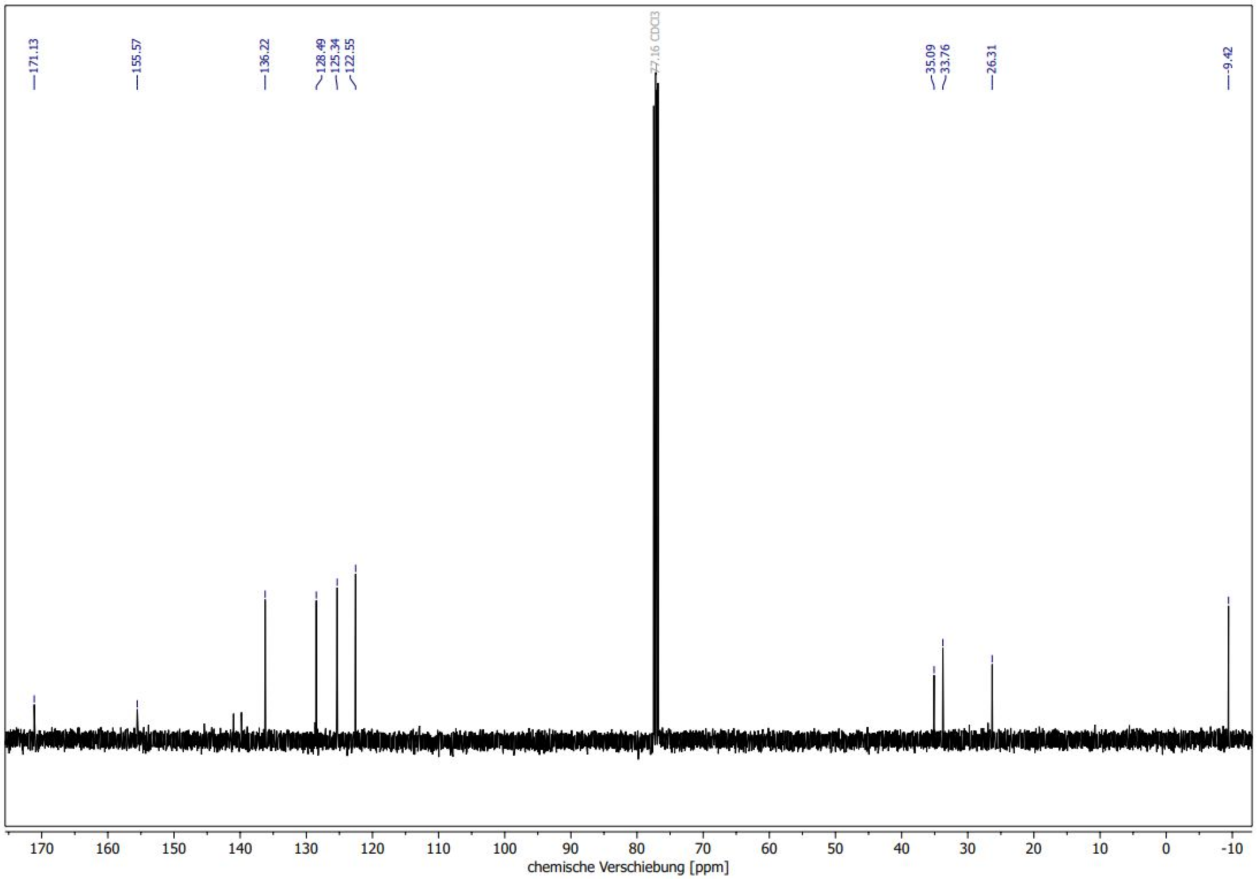

^{119}Sn NMR spectrum

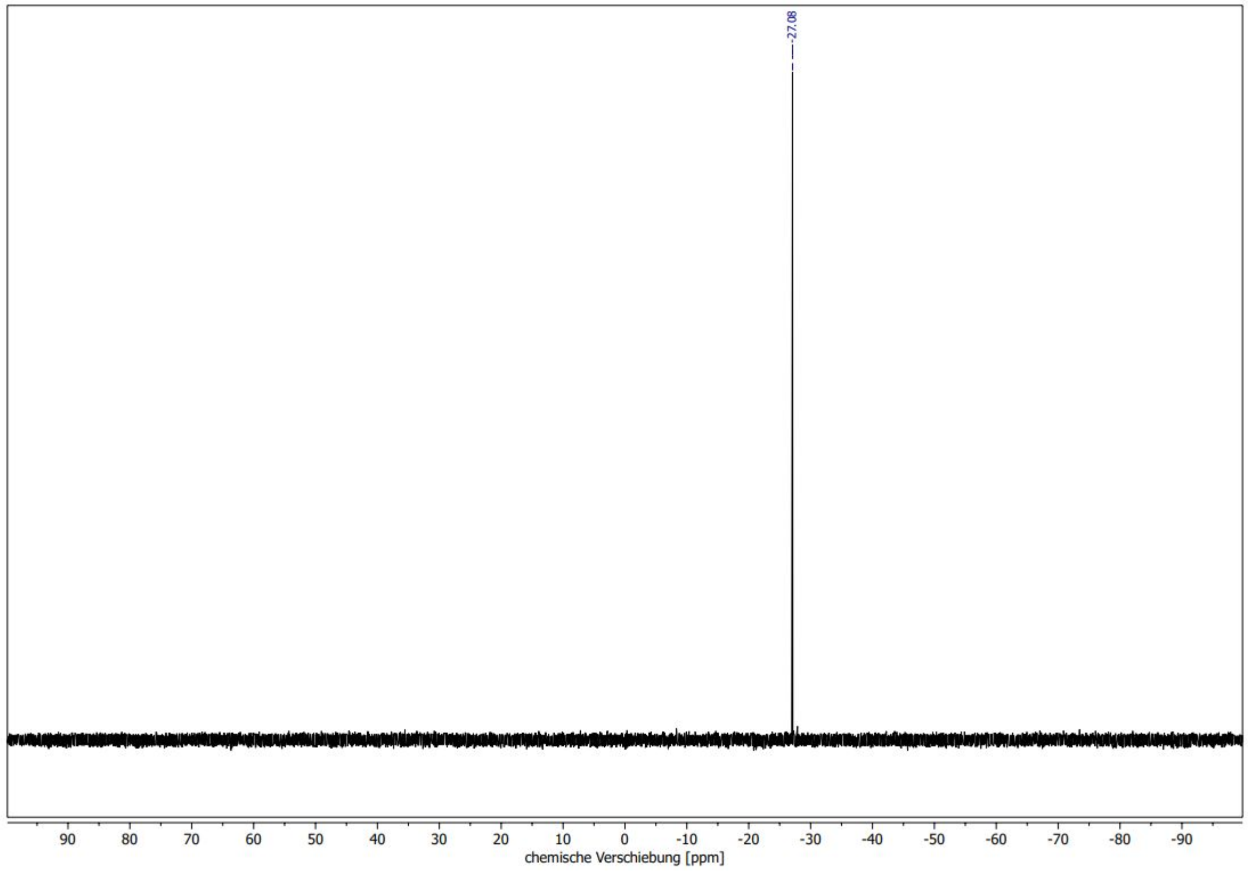

Mass spectra of compounds

*HR mass spectrum (ESI+) of compound **8***

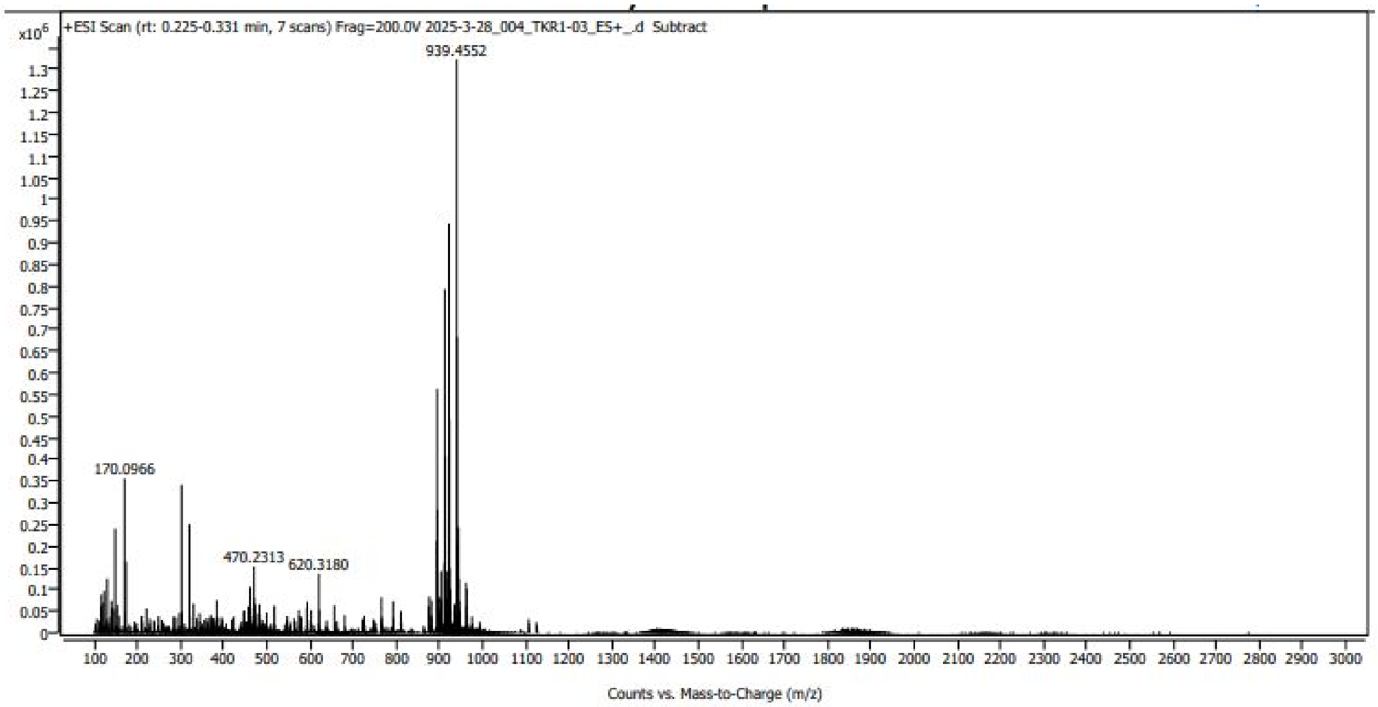

*HR mass spectrum (ESI+) of compound **9***

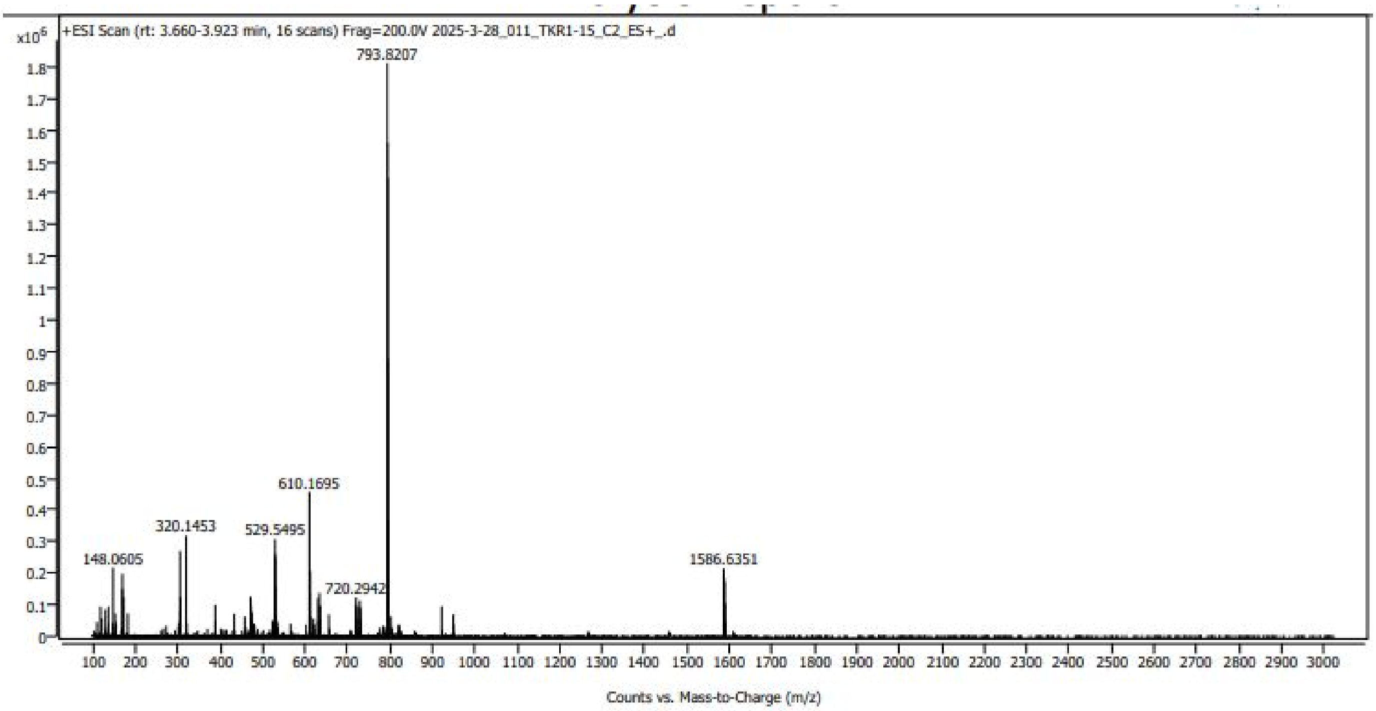

HR mass spectrum (ESI+) of compound ***11***

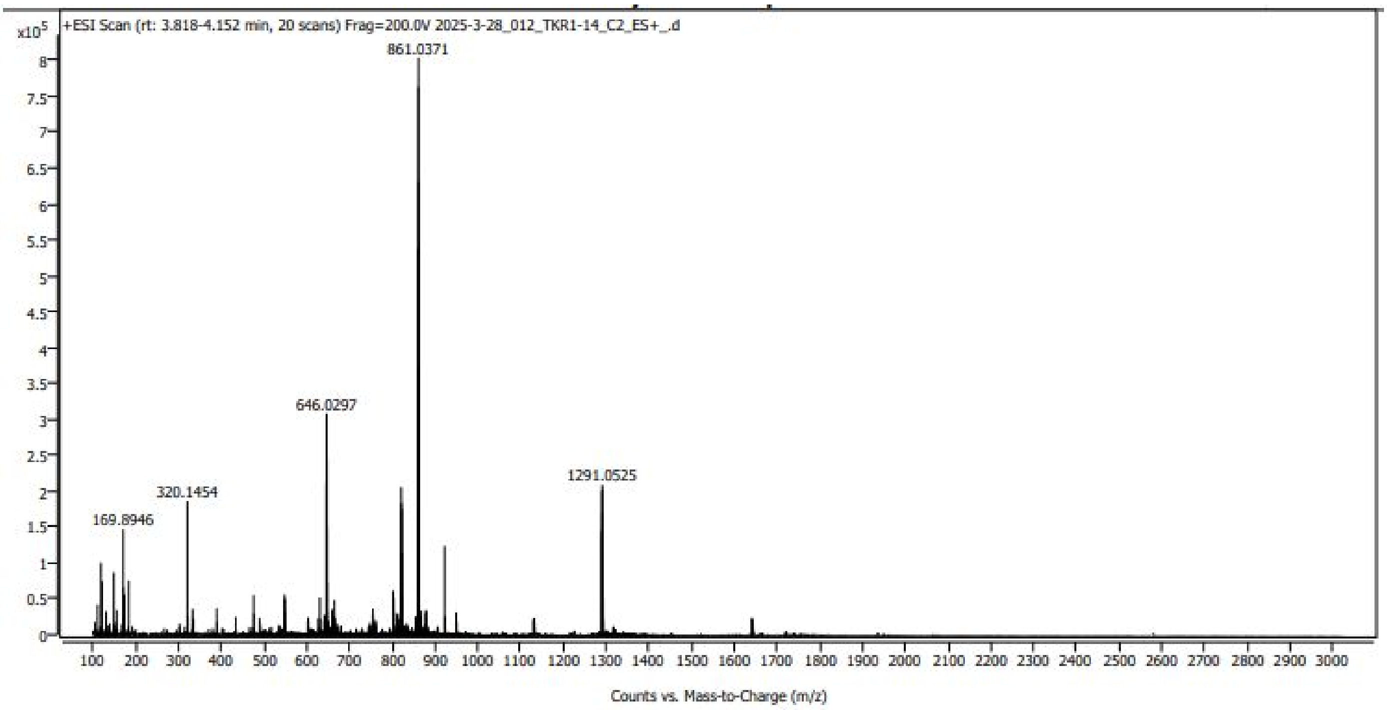

Spectrum Peaks

m/z	Z	Abund	Abund %	m/z (Calc)	Diff (ppm)	Ion Species	Formula	Ion Type
118.0863		100293	12.48					
148.0605		86813	10.80					
169.8946		147059	18.30					
320.1454		185774	23.12					
645.5288		210694	26.22					
645.7796	2	296891	36.95					
646.0297	2	307990	38.33					
646.2800	2	246048	30.62					
646.5305	2	150746	18.76					
820.8260	2	205576	25.59					
821.3275	2	183439	22.83					
821.8272	2	174629	21.73					
822.3279	2	113589	14.14					
860.3692		538949	67.08					
860.7035	1	762939	94.96					
861.0371	1	803470	100.00					
861.3708		631086	78.55					
861.7047	1	374246	46.58					
862.0387	1	180154	22.42					
922.0098		123516	15.37					
1290.0507	2	144916	18.04					
1290.5522	2	200788	24.99					
1291.0525	2	208586	25.96					
1291.5532	2	168608	20.98					
1292.0539	2	101572	12.64					

Mass spectrum (MALDI) of ***mcp-M-Sn-alb-PSMA (10)***

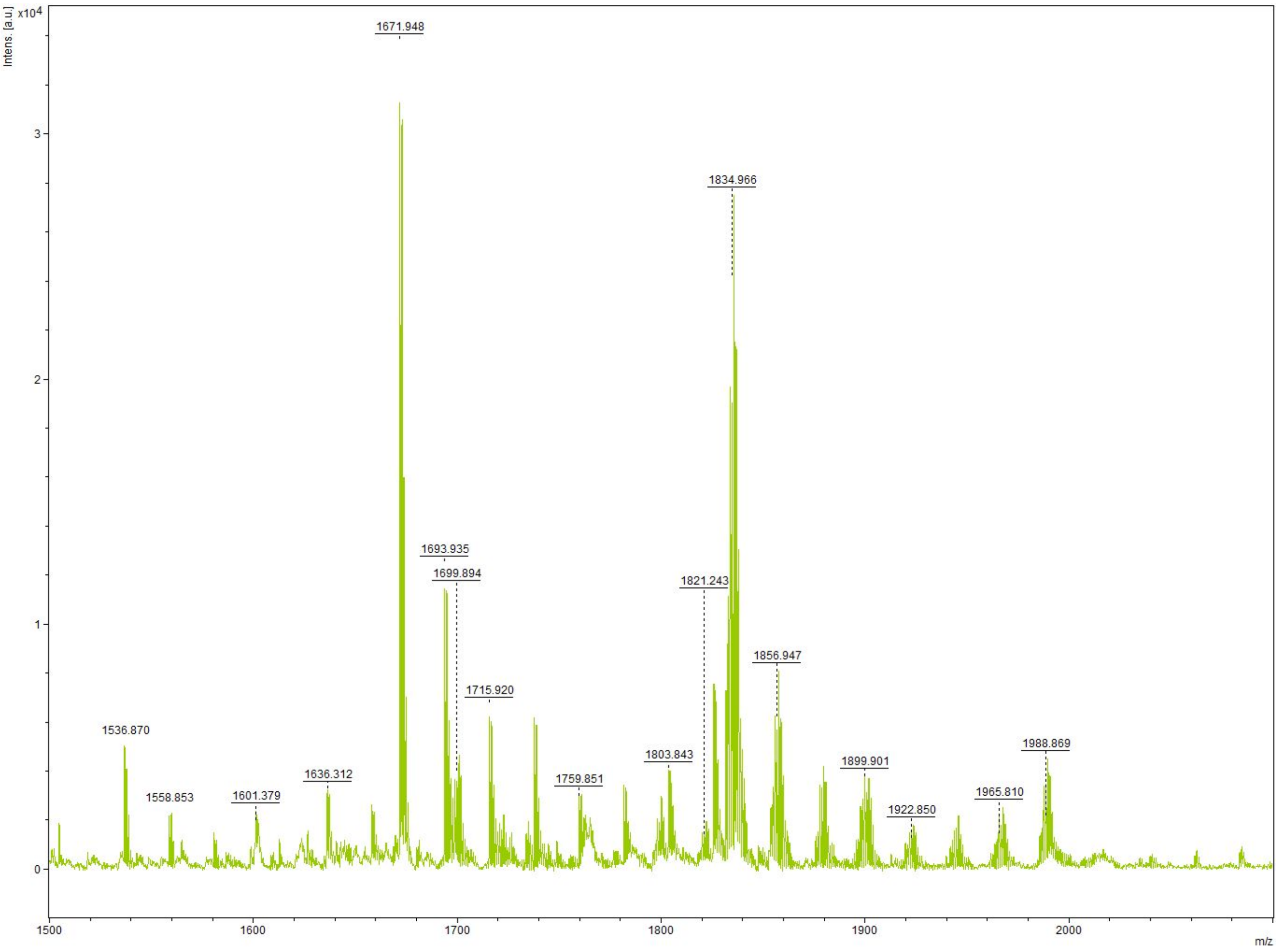

HR mass spectrum (ESI+) of ***mcp-M-Sn-alb-PSMA (10)***

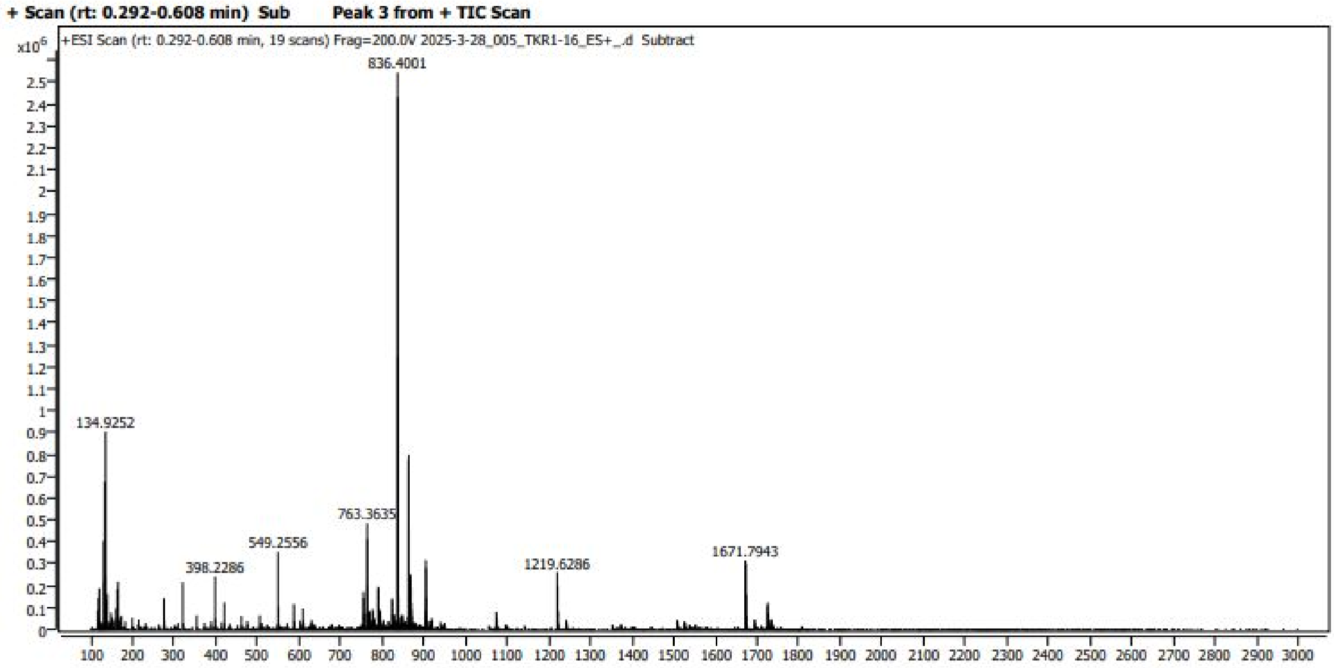

*Mass spectrum (MALDI) of **mcp-D-Sn-alb-PSMA (12)***

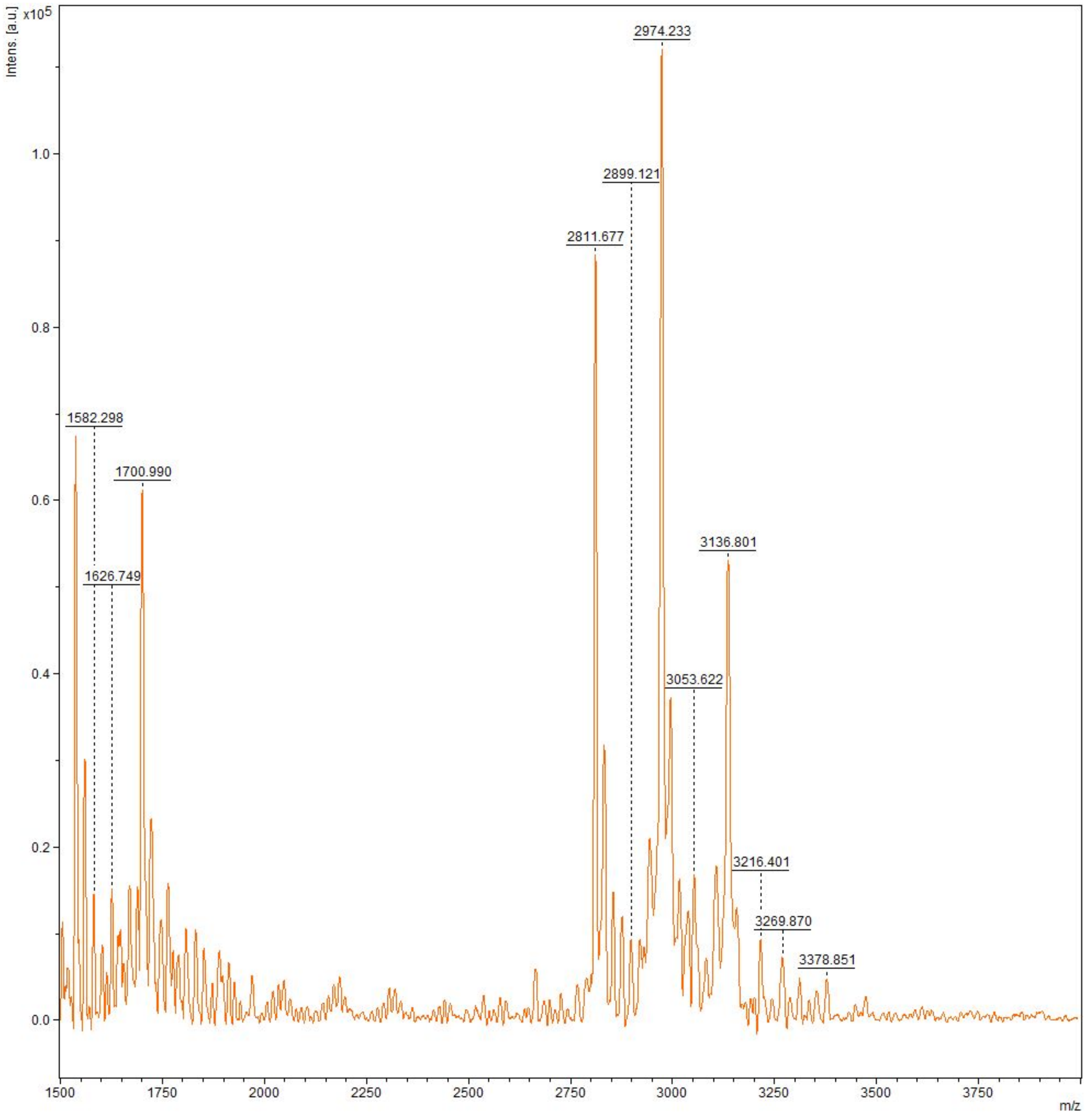

HR mass spectrum (ESI+) of ***mcp-D-Sn-alb-PSMA (12)***

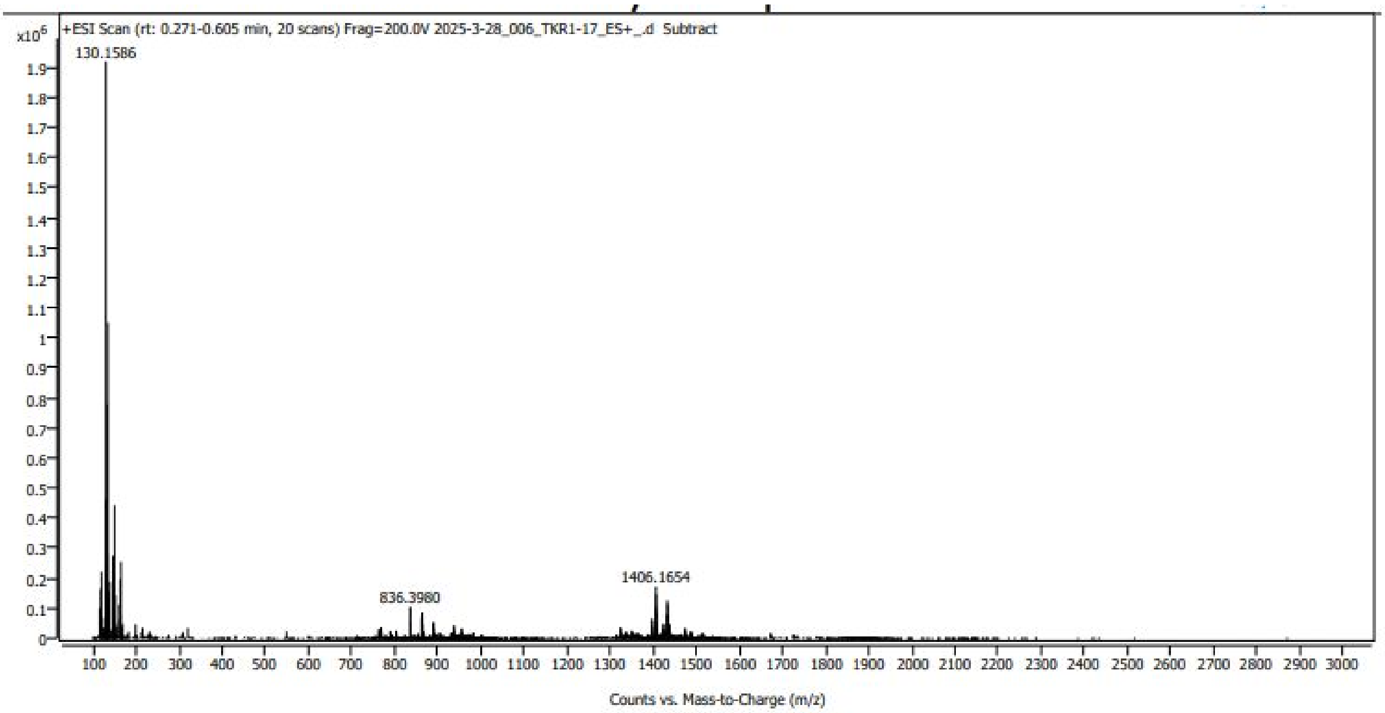

Radio-HPLC analysis

Radio-HPLC-chromatogram of ***mcp-M-[^{123}I]alb-PSMA***

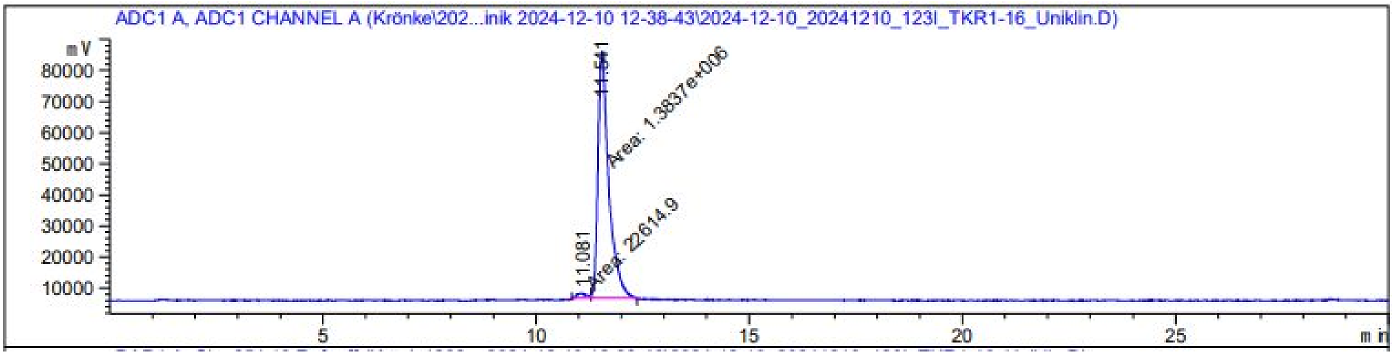

Peak #	RetTime [min]	Type	Width [min]	Area [mV*s]	Height [mV]	Area %
1	11.081	MF	0.2591	2.26149e4	1454.68079	1.6081
2	11.541	FM	0.2913	1.38370e6	7.91570e4	98.3919
Totals :				1.40632e6	8.06117e4	

Radio-HPLC-chromatogram of ***La-mcp-M-[^{123}I]alb-PSMA***

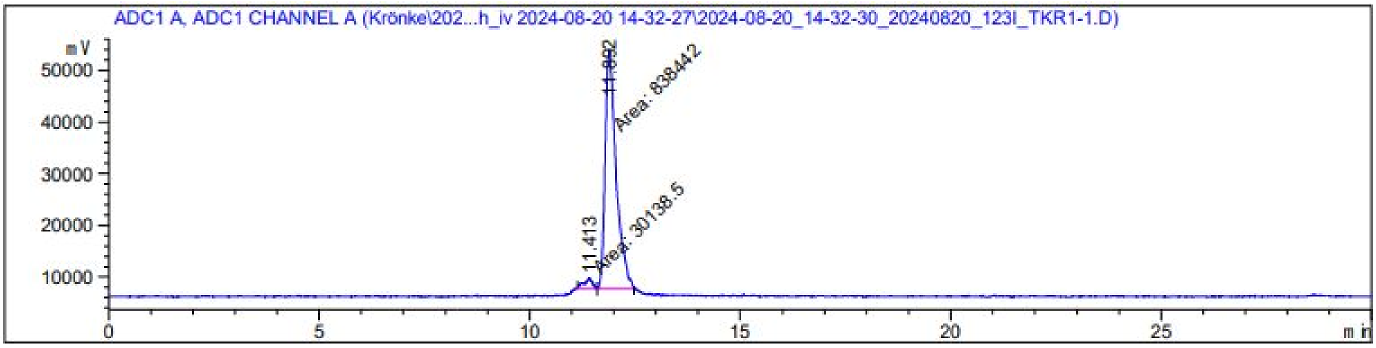

Peak #	RetTime [min]	Type	Width [min]	Area [mV*s]	Height [mV]	Area %
1	11.413	MF	0.2304	3.01385e4	2180.56250	3.4699
2	11.892	FM	0.3028	8.38442e5	4.61547e4	96.5301
Totals :				8.68580e5	4.83353e4	

Radio-HPLC-chromatogram of ***mcp-D-[***^{123}***I]alb-PSMA***

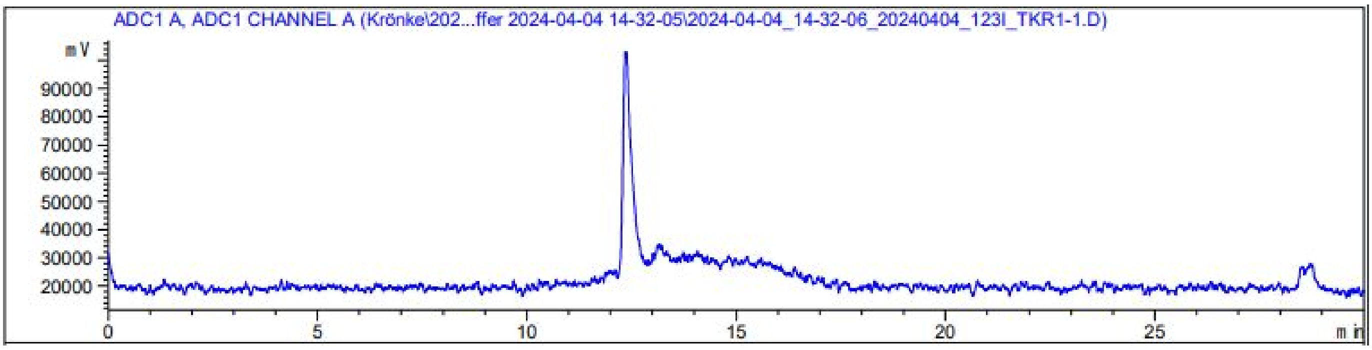

Supplement: Supplementary file 1 [file jm6c00161_si_001.pdf]
